# Thiosemicarbazone modified zeolitic imidazolate framework (TSC-ZIF) for mercury(ii) removal from water†

**DOI:** 10.1039/d1ra02025k

**Published:** 2021-04-30

**Authors:** Amani Jaafar, Carlos Platas-Iglesias, Rana A. Bilbeisi

**Affiliations:** American University of Beirut (AUB), Department of Civil and Environmental Engineering Riad El Solh Beirut 1107-2020 Lebanon rb102@aub.edu.lb; Centro de Investigacións Científicas Avanzadas (CICA) and Departamento de Química, Facultade de Ciencias, Universidade da Coruña 15071 A Coruña Galicia Spain

## Abstract

Zeolitic imidazolate frameworks (ZIF-8), and their derivatives, have been drawing increasing attention due to their thermal and chemical stability. The remarkable stability of ZIF-8 in aqueous and high pH environments renders it an ideal candidate for the removal of heavy metals from wastewater. In this study, we present the preparation of novel aldehyde-based zeolitic imidazolate frameworks (Ald-ZIF) through the integration of mixed-linkers: 2-methylimidazole (MIM) and imidazole-4-carbaldehyde (AldIM). The prepared Ald-ZIFs were post-synthetically modified with bisthiosemicarbazide (Bisthio) and thiosemicarbazide (Thio) groups, incorporating thiosemicarbazone (TSC) functionalities to the core of the framework. This modification results in the formation of TSC-functionalized ZIF derivatives (TSC-ZIFs). Thiosemicarbazones are versatile metal chelators, hence, adsorption properties of TSC-ZIFs for the removal of mercury(ii) from water were explored. Removal of mercury(ii) from homoionic aqueous solutions, binary and tertiary systems in competition with lead(ii) and cadmium(ii) under ambient conditions and neutral pH are reported in this study. MIM_3.5_:Thio_1_:Zn improved the removal efficiency of mercury(ii) from water, up to 97% in two hours, with an adsorption capacity of 1667 mg g^−1^. Desorption of mercury(ii) from MIM_3.5_:Thio_1_:Zn was achieved under acidic conditions, regenerating MIM_3.5_:Thio_1_:Zn for five cycles of mercury(ii) removal. TSC-ZIF derivatives, designed and developed here, represent a new class of dynamically functionalized adsorption material displaying the advantages of simplicity, efficiency, and reusability.

## Abstract 2

Zeolitic imidazolate frameworks Ald-ZIF were obtained by mixing two imidazole-based linkers with zinc(ii). Post-synthetically modified Ald-ZIFs with thiosemicarbazide group improved mercury(ii) removal efficiency from water at a capacity of 1667 mg g^−1^.

## Graphical abstract

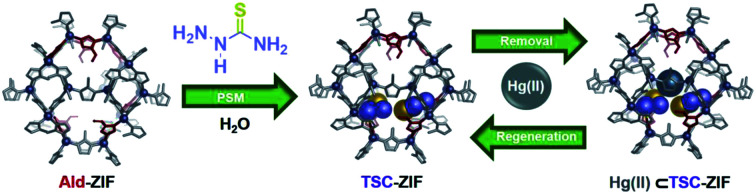

## Introduction

Metal–organic frameworks (MOFs) are a class of adsorbent materials composed of metal cations, connected to polytopic organic linkers *via* coordination bonds. MOFs,^1–3^ as porous crystalline materials, combine high porosity, large surface area, flexible pore size and shape,^4,5^ and in most cases, a high stability,^6^ with simple, economical and convenient direct synthetic routes.^7–9^ The porous structure exhibited by MOFs offers large surface areas, reaching ∼5200 m^2^ g^−1^,^10^ and variety of pore dimensions and topologies.^11^ All the before mentioned properties render MOFs suitable candidates for catalysis,^12–16^ separation,^17,18^ gas storage,^19–21^ and drug delivery^19,22–28^ among other applications.^16,29–32^

The flexibility of the coordination bond, joining the organic linker to metal ion, permits chemical modulations through post-synthetic modification (PSM) of the metal–organic framework. This promotes MOFs to high performance, tailor-made materials.^33^ PSM, ranging from carrying out chemical transformation^34–36^ or exchange on pre-synthesized materials,^37,38^ has emerged as a powerful method for functionalizing MOFs.^39,40^

Zeolitic imidazolate frameworks (mainly, ZIF-8) received much attention due to their thermal and chemical stability which makes them ideal candidates for further adjustment of their physical and chemical features to attain satisfactory performances in a wide range of potential applications.^41–43^ZIF-8 structures have been prepared using different approaches, mainly hydro and solvothermal.^44–46^ The remarkable stability of ZIF-8 in aqueous and high pH environments renders it an ideal candidate for the removal of heavy metals from wastewater.^47^

Heavy metals, in general, are toxic to all living organisms.^48^ Mercury, in particular, is considered to be extremely dangerous due to high solubility and bioaccumulation properties.^49,50^ Different techniques have been developed for the removal of heavy metals from contaminated wastewater,^51,52^ such as chemical precipitation,^53^ membrane filtration,^54^ electrochemical treatments,^55,56^ adsorption^57,58^ and ion exchange.^59,60^ Removal of mercury cations from contaminated wastewater has been recently achieved using novel sulfur-functionalized MOFs,^61–63^ adsorption parameters of these MOFs are presented in Table 1. Relevant parameters include maximum mercury adsorption capacity (mg g^−1^), retention time (minutes), and pH of the medium characterising HKUST-1,^62^ thiol-functionalized ZIF-90 (ZIF-90-SH),^64^ UiO-66-NHC(S)NHMe,^65^ FJI-H12 ^66^ and other robust MOFs^67^ are presented in the table. The most recent example of an efficient Hg(ii) adsorption material is using hybrid material – ZnS with ZIF-8 on filter paper. The high sulfur content in the hybrid material exhibits outstanding adsorption of Hg(ii), where the removal was achieved through simple filtration of contaminated water using the monolith ZnS-ZIF-8.^68^

**Table 1:** Comparison of maximum mercury(ii) adsorption capacity *q*_max_ (mg g^−1^), pH, and adsorption time (min) of TSC-ZIF (MIM_3.5_:Thio_1_:Zn) with previously reported sulfur-functionalized MOFs^a^

MOF	*q*_max_ (mg g^−1^)	pH	*t* (min)	Ref.
Thiol-HKUST-1	714	—	120	62
ZIF-90-SH	22	—	1440	64
UiO-66-NHC(S)R*	769	—	240	65
FJI-H12	440	7	60	66
Zr-MSA	734	5	5	67
ZnS-ZIF-8	925.9	5	<2	68
MIM_3.5_:Thio_1_:Zn	**1667**	**7**	**30**	**This study**

a R* = NHCH_3_.

**Scheme 1:**
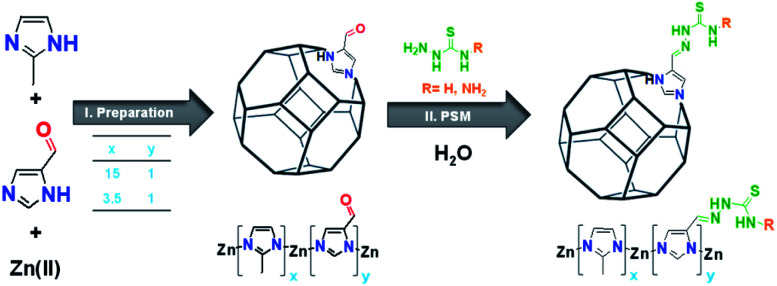
Schematic illustration of (I) preparation of the two Ald-ZIFs (MIM*_x_*:AldIM*_y_*:Zn) and (II) post-synthetic modification (PSM) of Ald-ZIFs to obtain TSC-ZIFs through the incorporation of thiosemicarbazone derivatives (bisthiosemicarbazone and/or thiosemicarbazone) in aqueous solution.

This study presents the preparation of a new class of aldehyde modified ZIF-8 derivatives (Ald-ZIF), which were further functionalized with thiosemicarbazone (TSC) groups for the removal of mercury(ii) ions from water. These Ald-ZIF were prepared through the integration of mixed-linkers: 2-methylimidazole (MIM) and imidazole-4-carbaldehyde (AldIM). The linkers were combined in two ratios (*x*_1_ = 15, *y*_1_ = 1 and *x*_2_ = 3.5, *y*_2_ = 1, where *x* and *y* represent the relative contents of MIM and AldIM, respectively) to yield two Ald-ZIF: MIM_15_:AldIM_1_:Zn and MIM_3.5_:AldIM_1_:Zn. The major component in all prepared Ald-ZIF is MIM, to retain the chemical and physical properties originally exhibited by ZIF-8. Incorporation of AldIM allows for further functionalization of the ZIF's framework, through post-synthetic modification (PSM). Accordingly, the prepared MIM*_x_*:AldIM*_y_*:Zn were post-synthetically modified with two thiosemicarbazide based functionalities; bis (NH_2_–NH–CS–NH–NH_2_) and thio (NH_2_–NH–CS–NH_2_) semicarbazones, through the condensation of the aldehyde (in AldIM) to bis/thiosemicarbazide. This successful PSM resulted in the formation of four new thiosemicarbazone zeolitic imidazole framework derivatives (TSC-ZIF), as demonstrated in Scheme 1.

## Results and discussion

Aldehyde modified ZIF-8 (Ald-ZIF) derivatives were successfully prepared through modifying the synthetic procedure of ZIF-8.^69^ Simultaneous incorporation of commercially available 2-methylimidazole (MIM) and imidazole-4-carbaldehyde (AldIM) in two different ratios (*x*_1_ = 15, *y*_1_ = 1 and *x*_2_ = 3.5, *y*_2_ = 1) yielded MIM*_x_*:AldIM*_y_*:Zn (please refer to the ESI†). MIM_15_:AldIM_1_:Zn was successfully prepared through hydrothermal conditions using Zn(OAc)_2_ in water/methanol at room temperature, whereas MIM_3.5_:AldIM_1_:Zn was only obtained under solvothermal conditions using Zn(NO_3_)_2_ in DMF at 110 °C for 72 h. Dynamic post-synthetic modification of MIM_15_:AldIM_1_:Zn and MIM_3.5_:AldIM_1_:Zn was performed on the aldehyde that condenses to the amine of the bisthiosemicarbazide (Bisthio, R = –NH_2_) or thiosemicarbazide (Thio, R = –H), yielding four novel TSC-ZIF: MIM_15_:Bisthio_1_:Zn, MIM_15_:Thio_1_:Zn, MIM_3.5_:Bisthio_1_:Zn and MIM_3.5_:Thio_1_:Zn (Scheme 1). The degree of functionalization of Ald-ZIFs was monitored by FTIR and NMR spectroscopies.

### Characterisation of Ald-ZIF and TSC-ZIF

#### FTIR measurements

A band at 1690 cm^−1^ corresponding to the stretching *ν*(C=O) vibration of the carbonyl group was observed in the IR spectra of MIM_15_:AldIM_1_:Zn and MIM_3.5_:AldIM_1_:Zn. This band disappears upon introducing the TSC-functionalities, indicating successful post synthetic modification of Ald-ZIF. The conversion of the aldehyde groups, in Ald-ZIFs, to imine groups in TSC-ZIFs, was further confirmed by the strong band at 1604 cm^−1^ corresponding to the C=N stretching vibration.^70^ Two new IR bands are also observed at 1047 and (1864) cm^−1^, indicative of the presence of the thiosemicarbazone group corresponding to the *ν*(C–N) and *ν*(C=S) stretching vibrations, respectively (Fig. S1 in the ESI†). Since the linker contains a thioamide –NH–C=S functional group, it can exhibit the thione-thiol tautomerism.^71^ The thiol *ν*(S–H) band around 2570 cm^−1^ is absent from the IR spectra of the TSC-ZIFs, while the *ν*(N–H) band is present at 3153 cm^−1^, indicating that, in the solid-state, the linker remains as the thione tautomer. The proposed IR assignments of the ZIFs are in good agreement with literature data.^72–74^ The introduction of the thioamide groups in MIM_3.5_:Bisthio_1_:Zn and MIM_3.5_:Thio_1_:Zn resulted in new vibrational bands, with characteristic absorptions at 2122 cm^−1^ corresponding to the *ν*_as_(NH–C=S) modes.^71^

#### NMR analysis

The degree of functionalization of Ald-ZIFs and TSC-ZIFs was determined by digesting the ZIFs under acidic conditions. The imine bond (linking the AldIM and the bisthio/thiosemicarbazide) does not get affected by the acidic conditions. This adopted method follows the general trend of cleaving MOFs, where the disassembly of the MOF takes place without cleaving the imine bond.^75^ Nuclear Magnetic Resonance (NMR) measurements were performed after digesting ZIF-8, Ald-ZIFs, and TSC-ZIFs in 80% deuterated solvent (DMSO-d_6_ or D_2_O-d_2_) mixed with 20% d_4_-acetic acid (CD_3_COOD). The chemical shifts of both imidazole linkers, 2-methylimidazole (MIM) and imidazole-4-carbaldehyde (AldIM), were referenced to DMSO-d_6_ for MIM_15_:AldIM_1_:Zn, MIM_15_:Bisthio_1_:Zn, and MIM_15_:Thio_1_:Zn, whereas D_2_O-d_2_ was used for referencing ZIF-8, MIM_3.5_:AldIM_1_:Zn, MIM_3.5_:Bisthio_1_:Zn and MIM_3.5_:Thio_1_:Zn (see Fig. S2–S11†). The stoichiometry of the two imidazole linkers in the hybrid MIM*_x_*:AldIM*_y_*:Zn structures were determined by integrating the areas under the peak of the methyl protons of 2-methylimidazole and the aldehyde proton of imidazole-4-carbaldehyde (^1^H NMR spectra in Fig. S4 and S7†). The carbonyl resonance of the AldIM, was also apparent at 183 ppm in ^13^C NMR of the digested MIM_3.5_:AldIM_1_:Zn (^13^C NMR spectra in Fig. S8†).

New sets of peaks were observed in the obtained NMR spectra of MIM*_x_*:Bisthio*_y_*:Zn and MIM*_x_*:Thio*_y_*:Zn; including a new peak in the aromatic range representative for the formation of the imine group HC=N, consistent with successful functionalization of the carbonyl group of AldIM with the bis/thiosemicarbazone groups. The ^1^H NMR resonances of MIM_3.5_:Thio_1_:Zn correspond to the imine proton at 8.1 ppm and the three amine groups at 8.0, 7.4 and 7.9 ppm (see Fig. S10†). ^13^C NMR spectra of MIM_3.5_:Bisthio_1_:Zn and MIM_3.5_:Thio_1_:Zn exhibit two peaks at 142.03 and 178.07 ppm attributable to the C=N and C=S groups, respectively. The total transformation of the carbonyl groups in MIM*_x_*:AldIM*_y_*:Zn to bis/thiosemicarbazone groups was demonstrated by the absence of the aldehyde proton peak at 9.69 and 9.17 ppm, indicating a nearly complete conversion of post-synthetic modification. This was further confirmed by disappearance of the ^13^C NMR peak at 183 ppm, corresponding to the carbonyl group of the parent MIM_3.5_:AldIM_1_:Zn, in TSC-ZIFs (Fig. S9 and S11†).

#### Powder X-ray diffraction (PXRD) measurements

**Fig. 1:**
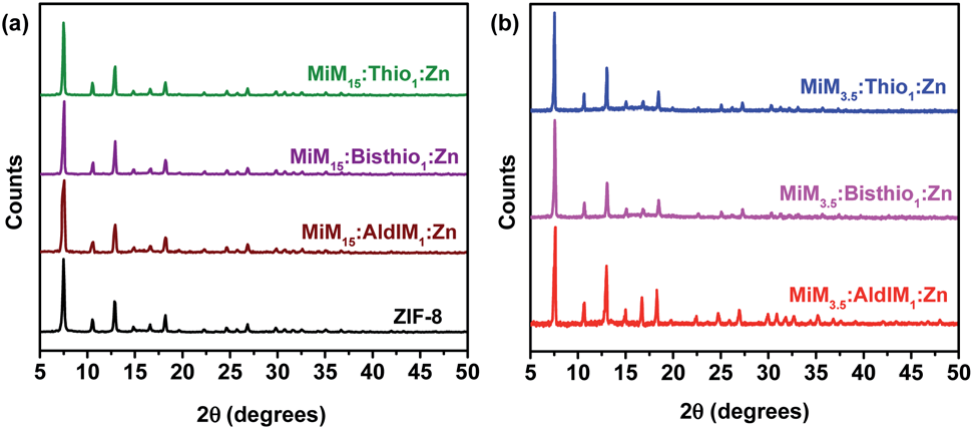
PXRD pattern of the two ratios of: (a) MIM_15_:AldIM_1_:Zn, (b) MIM_3.5_:AldIM_1_:Zn.

Crystallinity pattern and cubic framework structure of ZIF-8 was retained in Ald-ZIFs and TSC-ZIFs, as indicated by their PXRD diffraction data (the consistent peak positions and relative intensities as displayed in Fig. 1).^12^ The PXRD diffraction patterns of the hybrid Ald-ZIF and TSC-ZIF match the diffraction patterns of the single-linker ZIF-8 structures, with all ZIFs exhibiting virtually identical cubic unit cells. Furthermore, XRD details of the reported ZIFs indicate that all samples have relatively the same framework topology with small differences in electron density and lattice constant.

The prominent reflections at 2*θ* = 7.4°, 12.7° and 18.0° for the resulting ZIFs are clear, and are in good agreement with the simulated patterns for ZIF-8 using single crystal data (Fig. S12†), with a typical SOD structure.^68^

#### N_2_ sorption–desorption isotherm

The dinitrogen sorption isotherms of the Ald-ZIFs and TSC-ZIFs were measured at 77 K, and the Brunauer–Emmett–Teller (BET) and pore volume of all the samples were calculated (Table 2 and Fig. S13†). ZIF samples were degassed overnight at 423 K before surface area determination. All ZIF samples, including the parent ZIF-8, were analysed using the same protocol since sorption behaviour for ZIFs is sensitive to handling and pre-treatment procedures.

**Table 2:** Textural parameters and the yields of the ZIF-8, Ald-ZIFs and the TSC-ZIFs

Sample	*S*_BET_ a (m^2^ g^−1^)	*V*_micro_ b (cm^3^ g^−1^)	Yield (%)
ZIF-8	1555	0.73	93
MIM_15_:AldIM_1_:Zn	1396	0.63	75
MIM_15_:Bisthio_1_:Zn	1128	0.5	40
MIM_15_:Thio_1_:Zn	1237	0.58	77
MIM_3.5_:AldIM_1_:Zn	1130	0.37	54
MIM_3.5_:Bisthio_1_:Zn	623	0.20	57
MIM_3.5_:Thio_1_:Zn	679	0.26	62

a *S*_BET_ is the BET surface area.

b *V*_micro_ is the *t*-plot micropore volume.

As demonstrated in Table 2, the calculated BET surface area for ZIF-8 is 1555 m^2^ g^−1^, matching reported values in the literature (1580 m^2^ g^−1^). Given that the degree of post-synthetic modification and the size of the substituents dictate the available volume for the dinitrogen adsorption within the ZIF,^37,76^ we expected the BET surface area and pore volume to decrease in the mixed-linker ZIFs, relative to ZIF-8. Indeed, all the mixed-linker ZIFs exhibit lower surface areas, with the higher aldehyde incorporation (MIM_3.5_:AldIM_1_:Zn) showing a more significant reduction in surface area than the lower aldehyde incorporation species (MIM_15_:AldIM_1_:Zn). Thus, the BET surface area of MIM_15_:AldIM_1_:Zn was found to be 1397 m^2^ g^−1^, marginally lower than that of ZIF-8. Whereas, the surface area of MIM_15_:Bisthio_1_:Zn and MIM_15_:Thio_1_:Zn is reduced relative to that of MIM_15_:AldIM_1_:Zn due to the decrease of internal void space associated with the introduction of the carbonyl groups. Similarly, post-synthetic modification of MIM_3.5_:AldIM_1_:Zn results in a more significant decrease in BET surface area to 623 and 679 m^2^ g^−1^ for MIM_3.5_:Bisthio_1_:Zn and MIM_3.5_:Thio_1_:Zn, respectively. This can be attributed to the higher degree of modification with bisthiosemicarbazone and thiosemicarbazone groups.

#### SEM-EDX measurements

**Fig. 2:**
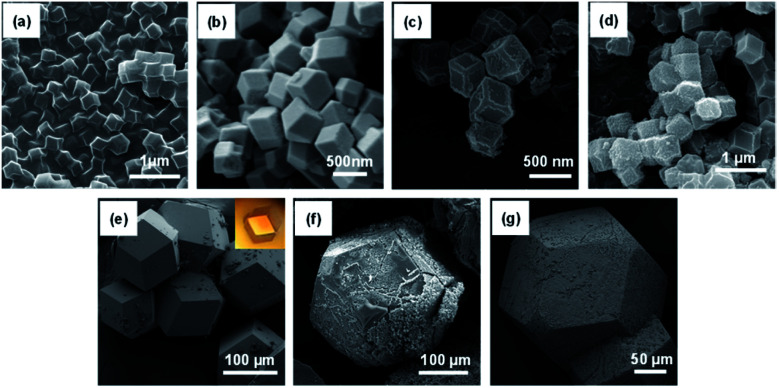
SEM images for (a) ZIF-8, (b) MIM_15_:AldIM_1_:Zn, (c) MIM_15_:Bisthio_1_:Zn, (d) MIM_15_:Thio_1_:Zn, (e) MIM_3.5_:AldIM_1_:Zn (inset: crystals of MIM_3.5_:AldIM_1_:Zn), (f) MIM_3.5_:Bisthio_1_:Zn and (g) MIM_3.5_:Thio_1_:Zn.

Surface morphology and chemical composition of Ald-ZIFs and TSC-ZIFs were also investigated using SEM (Fig. 2) and EDX (Fig. S14†). The crystals of original ZIF-8 and MIM_15_:AldIM_1_:Zn present cubic and rhombic dodecahedral shapes, respectively. Both exhibited smooth surfaces, and an average size of 500 nm. However, the surfaces of the MIM_15_:Bisthio_1_:Zn sample experience a significant morphological change, as the shape changed from a rhombic dodecahedron with smooth faces and sharp edges in MIM_15_:AldIM_1_:Zn, to truncated-edge rhombic dodecahedra for MIM_15_:Bisthio_1_:Zn, but the size of the particles does not change significantly (∼500 nm). The crystals of MIM_15_:Thio_1_:Zn are rhombic dodecahedra with a bumbled surface due to the substitution of thiosemicarbazone. The crystals of MIM_3.5_:AldIM_1_:Zn, MIM_3.5_:Bisthio_1_:Zn and MIM_3.5_:Thio_1_:Zn are rhombic dodecahedral with different aspects, smooth on the surface and a large size up to 100 μm (Fig. 2).

The EDX spectra of the TSC-functionalized ZIFs confirmed that the ZIF samples are composed of C, N, O, Zn, and S, as presented in Fig. S14.† The relative content of S in the functionalized TSC-ZIFs were determined by EDX spectra.

#### TGAs curves analysis

Thermal stability of the prepared Ald-ZIF and TSC-ZIF samples, relative to ZIF-8, was characterised by thermal gravimetric analysis, (TGA) (Fig. S15†). Prepared Ald-ZIFs and TSC-ZIFs display relatively high thermal stability similar to that of ZIF-8. MIM_3.5_:AldIM_1_:Zn undergoes an initial weight loss at about 450 °C, which can be attributed to the loss of carbonyl groups of the framework. A further weight loss at 550 °C is observed for ZIF-8 and MIM_3.5_:AldIM_1_:Zn due to framework decomposition. MIM_3.5_:Bithio_1_:Zn and MIM_3.5_:Thio_1_:Zn undergo weight loss at around 220 °C, which is not present in the ZIF-8 and Ald-ZIF samples. This can be attributed to the decomposition of the bisthiosemicarbazone and thiosemicarbazone groups, respectively. However, MIM_15_ : Bithio_1_:Zn and MIM_15_:Thio_1_:Zn exhibit negligible percentage weight loss at this temperature due to the low percentage of the TSC-linker within the framework of the ZIF.

#### DFT calculations

**Fig. 3:**
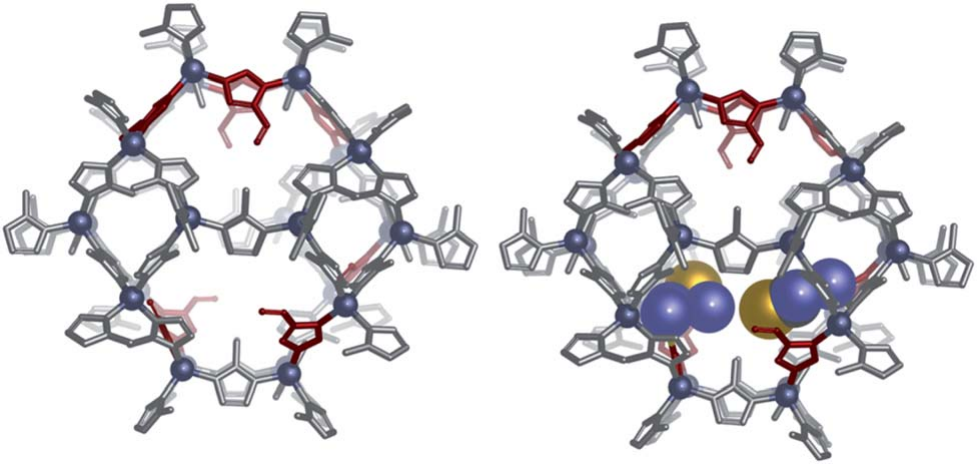
Models of the ZIF-8 structure incorporating (I) imidazole-4-carbaldehyde Ald-ZIF (left) and (II) imidazole-4-carbaldehyde and thiosemicarbazone TSC-ZIF (right) groups, optimized at the b3lyp/6-31G(d,p) level.

X-ray diffraction studies show that the incorporation of imidazole-4-carbaldehyde to the framework of ZIF-8 does not alter significantly the structure of the ZIF. To get insight into the orientation of the imidazole-4-carbaldehyde (AldIM) and thiosemicarbazone group (Thio) within the structure of the ZIF, we performed DFT calculations at the b3lyp/6-31G(d,p) level.^77–79^ The X-ray crystal structure of ZIF-8 was truncated to include 24 Zn(ii) ions that define the large cage of the structure, with 8 of the 60 2-methylimidazole (MIM) (supposed to be 1 to 3.5) groups being replaced by imidazole-4-carbaldehyde. These calculations yielded the expected tetrahedral coordination of the Zn ions provided by the bridging imidazole groups, with Zn–N distances of 2.0–2.04 Å (1.97 Å in the X-ray structure).^80^ Our DFT studies suggest that the carbaldehyde groups point inwards the six-membered hexagonal Zn rings, with the O atom being placed slightly below the mean plane defined by the six Zn ions (*ca.* 0.78 Å, Fig. 3). Indeed, changing the orientation of one of the aldehyde groups of this model towards one of the pores, defined by four ZnN_4_ tetrahedra, results in a significant increase in energy of 7.8 kJ mol^−1^. Subsequent calculations on the same model where two imidazole-4-carbaldehyde groups are replaced by thiosemicarbazone units suggest that the bulky thiosemicarbazone groups are also directed towards the large central pores of the structure.

### Mercury(ii) removal efficiency from water

The ability of the Ald-ZIF and TSC-ZIF derivatives to sequester mercury(ii) from aqueous solutions was investigated at ambient conditions (room temperature and neutral pH). Adsorption studies were conducted over a wide range of known mercury concentrations (ppm), with the change in the adsorbent colour (yellow crystals in the case of MIM_3.5_:Thio_1_:Zn) to black at high mercury(ii) concentrations serving as a preliminary indication of adsorption (Fig. S16†).

**Fig. 4:**
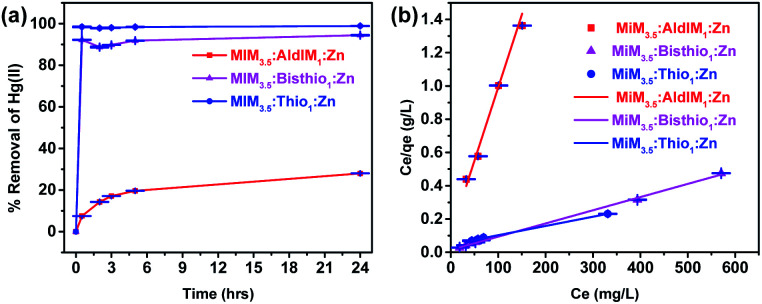
(a) Percentage removal of [Hg(ii)] with time using MIM_3.5_:AldIM_1_:Zn, MIM_35_:Bisthio_1_:Zn and MIM_3.5_:Thio_1_:Zn ([Hg(ii)] = 400 mg L^−1^), (b) Langmuir equation fitting curve for the adsorption isotherms for the MIM_3.5_:AldIM_1_:Zn, MIM_3.5_:Bisthio_1_:Zn and MIM_3.5_:Thio_1_:Zn.

Equations eqn (S1) and (S2)† were used to calculate the metal removal (%) from an aqueous solution where *C*_i_ and *C*_e_ represent the initial and equilibrium metal ion concentrations (mg L^−1^), respectively. The results for treating Hg(ii) solutions with ZIF-8, Ald-ZIF and TSC-ZIF derivatives are presented in Fig. 4(a) and S17.† Treatment of a 100 mg L^−1^ aqueous Hg(ii) solution with MIM_15_:Thio_1_:Zn and MIM_15_:Bithio_1_:Zn led to a 92.0% and 91.8% reduction in Hg(ii) content within 30 min at ambient conditions. However, treating a Hg(ii) solution of the same concentration, and under the same conditions, with ZIF-8 and MIM_15_:AldIM_1_:Zn resulted in 15% and 12% reduction, respectively (Fig. S17†). An obvious increase in the adsorptive removal of mercury cation was observed in TSC-ZIFs incorporating a higher degree of functionality (ratio *X*_2_ = 3.5: *y*_2_ = 1). Indeed, the treatment of a Hg(ii) solution (*C*_i_ = 400 mg g^−1^) with MIM_3.5_:Thio_1_:Zn and MIM_3.5_:Bithio_1_:Zn resulted in 98.9% and 94.4% removal of the Hg(ii) ion, respectively, with an unprecedented adsorption capacity (*q*_m_) of 1667 mg g^−1^ and 1250 mg g^−1^. This suggests that TSC-ZIFs possess both a high adsorption capacity and adsorption efficiency for the removal of mercury cations from water, in less than 2 hours and at ambient conditions (Fig. 4).

#### Adsorption isotherms for mercury(ii) removal from water

The Langmuir (eqn (S3) and (S4)†), and Freundlich (eqn (S5)†) adsorption models were applied to analyse the obtained adsorption data for TSC-ZIFs. The experimental data fit well the Langmuir equilibrium adsorption isotherm with a correlation coefficient of *R*^2^ > 0.99 (Fig. S18,†4(b) and Table 3). However, the fitted Freundlich model resulted in a lower correlation coefficient (*R*^2^ = 0.92, Table S1†) indicating that the adsorption process, follows a spontaneous single-layer chemical adsorption.^67,81^

**Table 3:** Langmuir adsorption isotherm fitting parameters for ZIF-8, Ald-ZIFs and TSC-ZIFs

Langmuir adsorption model parameters				
Samples	*K*_L_ (L mg^−1^)	*R*_L_	*q*_m_ (mg g^−1^)	*R* ^2^
ZIF-8	0.019	0.38	3	0.9995
MIM_15_:AldIM_1_:Zn	0.036	0.2	13.4	0.9943
MIM_15_:Bisthio_1_:Zn	0.59	0.017	128	0.9961
MIM_15_:Thio_1_:Zn	0.15	0.062	152	0.9969
MIM_3.5_:AldIM_1_:Zn	0.052	0.15	124	0.9934
MIM_3.5_:Bisthio_1_:Zn	0.053	0.02	1250	0.9987
MIM_3.5_:Thio_1_:Zn	0.013	0.07	1667	0.9962

The maximum adsorption capacities of ZIFs reported in this study are presented in Table 3. The separation factor (*R*_L_) was calculated to be between 0 and 1, indicating favourable adsorption of mercury cations into the prepared ZIFs (see Table 3). This can be attributed to the soft sulfur donor atoms incorporated in two different ratios within the pores of ZIF structures. In particular, the incorporation of bisthiosemicarbazone and thiosemicarbazone containing groups enhances mercury extraction performance with respect to the parent ZIF-8. Adsorption capacity of MIM_3.5_:Thio_1_:Zn exceeds the values recently reported for porous functionalized ZIFs (see Table 1).^64,65,67,68^

#### Adsorption kinetics

In order to evaluate the kinetic mechanism controlling the adsorption process, the effect of contact time between Hg(ii) and the adsorbents on the adsorption process was investigated.

The kinetic data were successfully fitted (Fig. S19 and S20†) to the pseudo-second-order kinetic model (eqn (S6)†), as indicated by the high correlation coefficient values (*R*^2^ > 0.99 for ZIF-8, MIM_15_:AldIM_1_:Zn, MIM_15_:Bisthio_1_:Zn, MIM_3.5_:AldIM_1_:Zn, MIM_3.5_:Bisthio_1_:Zn and *R*^2^ = 1 for MIM_15_:Thio_1_:Zn, MIM_15_:Thio_1_:Zn). Adsorption rate constants *k*_2_ at room temperature and neutral pH were determined to be 0.000059 < 0.00055 < 0.0032 g mg^−1^ min^−1^ for MIM_3.5_:AldIM_1_:Zn, MIM_3.5_:Bisthio_1_:Zn and MIM_3.5_:Thio_1_:Zn respectively (Table S2†).

The adsorption rate constant (*k*_2_ = 0.32 × 10^−2^ g mg^−1^ min^−1^) of MIM_3.5_:Thio_1_:Zn exceeds many other reported porous absorbents in the literature.^65^ This can be attributed to the higher degree of thiosemicarbazone incorporation. The steric demands of the thiosemicarbazone group occupying the inner surface of the pores and the high density of MIM_3.5_:Thio_1_:Zn adsorption sites give this TSC-ZIF the best performance.

#### Characterisation of TSC-ZIFs after removal of mercury(ii)

PXRD patterns of MIM_3.5_:Thio_1_:Zn did not change after the adsorption of Hg(ii) (Fig. 6(b)).The co-existence of Hg(ii) with the TSC-ZIFs is observed in SEM images (Fig. S21†) and EDX analysis (Fig. S22†). Similarly, TGA measurements of the Hg(ii) adsorbed onto MIM_3.5_:Thio_1_:Zn show one mass loss step at about 350 °C. This temperature is higher than that observed for MIM_3.5_:Thio_1_:Zn, confirming that the adsorbent maintained a stable framework structure after the adsorption process (Fig. S23†).

#### Competitive binding (binary and tertiary systems)

To evaluate the selectivity of MIM_3.5_:Thio_1_:Zn for Hg(ii) ion adsorption, we performed experiments in the presence of Pb(ii) and Cd(ii) as potential interfering species.

#### Binary adsorption with Pb(ii)

**Fig. 5:**
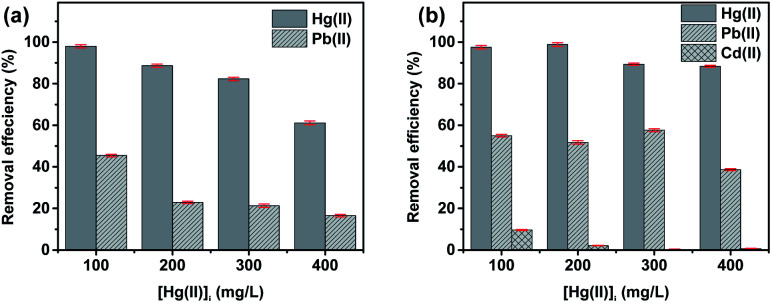
(a) Mercury(ii) adsorption onto MIM_3.5_:Thio_1_:Zn, binary system with [Pb(ii)]/[Hg(ii)]_i_ range: 100–400 mg L^−1^ and the initial [Pb(ii)] = 1000 mg L^−1^ in all samples tested, (b) mercury(ii) adsorption onto MIM_3.5_:Thio_1_:Zn in tertiary system with lead(ii) and cadmium(ii)/[Hg(ii)] range: 100–400 mg L^−1^ and the initial [Pb(ii)]_i_ = [Cd(ii)]_i_ = 1000 mg L^−1^ in all samples tested.

Binary metal containing systems were prepared using a fixed concentration of [Pb(ii)] = 1000 mg L^−1^ and a mercury concentration [Hg(ii)] ranging from 100 to 400 mg L^−1^. The percentage removal of both metal ions, existing in the binary system, is presented in Fig. 5(a). As depicted in the figure, MIM_3.5_:Thio_1_:Zn exhibits high removal efficiency for Hg(ii) and low removal for Pb(ii) ions, demonstrating a higher selectivity for Hg(ii). Meanwhile, at higher mercury cations concentration, a co-adsorption induces a decrease in its adsorption. Previous studies have explained the removal of the metal ions in the competitive adsorption system is based on the comparative assessment of their initial adsorption rates.^82,83^

#### Tertiary system with Pb(ii) and Cd(ii)

The concentration of Pb(ii) and Cd(ii) ions in the mixed solution was set each to 1000 mg L^−1^ and the [Hg(ii)] ranging from 100 to 400 mg L^−1^. As illustrated in Fig. 5(b), interference of the two metal ions minimally disturbs the removal efficiency for Hg(ii) ions, given that MIM_3.5_:Thio_1_:Zn exhibits lower removal efficiency towards Cd(ii) and Pb(ii) ions. Analysis of the removal efficiency values revealed that the order of adsorption was Hg(ii) > Pb(ii) ≫ Cd(ii). Removal efficiency for Cd(ii) and Pb(ii) decreases when the concentration of [Hg(ii)] increases, which demonstrates the selective adsorption for Hg(ii). Besides, the presence of Cd(ii) in the tertiary solution enhanced the removal efficiency for Hg(ii) and Pb(ii) (Fig. 5(b)).

This selective adsorption for Hg(ii) ions can be attributed to the higher affinity of thiosemicarbazone groups for Hg(ii) compared to other metal ions.^67^

#### Regeneration of MIM_3.5_:Thio_1_:Zn

**Fig. 6:**
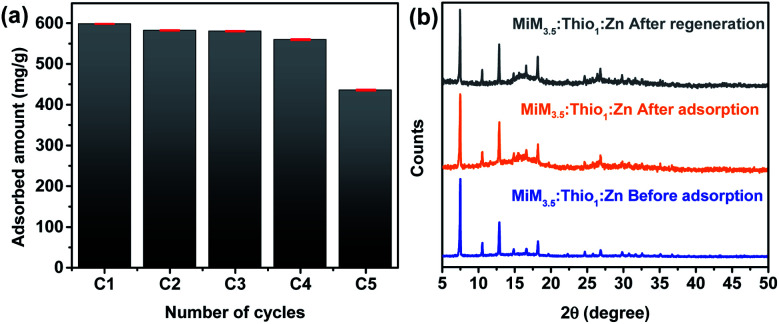
(a) Reusability of MIM_3.5_:Thio_1_:Zn for the adsorbed amount of Hg(ii) ([Hg(ii)]_i_ = 700 ppm) and (b) PXRD patterns of the MIM_3.5_:Thio_1_:Zn recorded before and after the adsorption of Hg(ii), and after the regeneration.

In actual applications, re-usability of adsorbents is crucial and reflects on the sustainability of the developed adsorbent. The reusability of MIM_3.5_:Thio_1_:Zn was assessed through cycles of regeneration of the ZIF in solution using *p*-toluene sulfonic acid (pH = 4) as a desorbent. Inspired by the literature, acidic conditions are expected to weaken the interaction between the adsorbate and adsorbent allowing for the regeneration of the TSC-ZIF.^68^ The relative efficiency of the removal of Hg(ii) in each through cycles of adsorption–desorption of mercury by MIM_3.5_:Thio_1_:Zn are presented in Fig. 6(a).

Over the five cycles, the amounts of mercury adsorbed decreased slightly with increasing the number of cycles, which might be caused by the loss of material during the recycling process. However, the adsorption efficiency was maintained at approximately 75% for the highest concentration of mercury(ii)/([Hg(ii)]_i_ = 700 ppm) of each cycle, indicating that MIM_3.5_:Thio_1_:Zn can be regenerated for cycles of mercury removal without compromising its removal efficiency. PXRD patterns of the recycled MIM_3.5_:Thio_1_:Zn (after the five cycle) were in good agreement with their PXRD patterns before adsorption (Fig. 6(b)). This demonstrates the high stability of the TSC-ZIF after the removal of mercury.

## Conclusions

A new class of aldehyde-based zeolitic imidazolate frameworks (Ald-ZIF) was developed to serve as a precursor, which can be modified for the removal of mercury cations from water. Bisthiosemicarbazone and thiosemicarbazone are the functional groups introduced, through post-synthetic modification, to the new class of Ald-ZIF resulting in the formation of four classes of TSC-ZIF derivatives. TSC-ZIF contain pendent thiosemicarbazone groups within the pores of the material. The degree of functionalization of Ald-ZIF was monitored using IR and NMR spectroscopies. Structural and thermal integrity of the TSC-ZIF were confirmed using PXRD studies, SEM-EDX and TGA analysis. The porosity of the TSC-ZIF derivatives (as measured using BET surface area calculations) are reduced relative to ZIF-8, depending on the degree of functionalization and size of introduced substituents. Sequestration of mercury(ii) from water at room temperature and neutral pH was achieved when treating Hg(ii) contaminated water with TSC-ZIF derivatives. Among TSC-ZIF derivatives, MIM_3.5_:Thio_1_:Zn showed the highest capacity for mercury(ii) ions due to the higher ratio of pore functionality, combined with the lower steric demands of the TSC group. Moreover, MIM_3.5_:Thio_1_:Zn showed selectivity for Hg(ii) in solutions containing competitive Pb(ii) and Cd(ii) metal ions. MIM_3.5_:Thio_1_:Zn was regenerated for up to four cycles of mercury(ii) removal without compromising the efficiency or structure of the ZIF. Therefore, TSC-ZIFs, as a new class of zeolitic frameworks, demonstrate promising adsorption capacity for heavy metals. Further work may provide a plethora of opportunities for generating new functionalized materials for other heavy metals and anions adsorption.

## Conflicts of interest

There are no conflicts to declare.

## Supplementary Material

RA-011-D1RA02025K-s001

## References

[cit1] Peng Y., Huang H., Zhang Y., Kang C., Chen S., Song L., Liu D., Zhong C. (2018). Nat. Commun..

[cit2] Bour J. R., Wright A. M., He X., Dincă M. (2020). Chem. Sci..

[cit3] Hou J., Sapnik A. F., Bennett T. D. (2020). Chem. Sci..

[cit4] Redfern L. R., Farha O. K. (2019). Chem. Sci..

[cit5] Su Z., Miao Y.-R., Zhang G., Miller J. T., Suslick K. S. (2017). Chem. Sci..

[cit6] Kalidindi S. B., Nayak S., Briggs M. E., Jansat S., Katsoulidis A. P., Miller G. J., Warren J. E., Antypov D., Corà F., Slater B., Prestly M. R., Martí-Gastaldo C., Rosseinsky M. J. (2015). Angew. Chem., Int. Ed..

[cit7] Stock N., Biswas S. (2012). Chem. Rev..

[cit8] Klet R. C., Wang T. C., Fernandez L. E., Truhlar D. G., Hupp J. T., Farha O. K. (2016). Chem. Mater..

[cit9] Howarth A. J., Liu Y., Li P., Li Z., Wang T. C., Hupp J. T., Farha O. K. (2016). Nat. Rev. Mater..

[cit10] Koh K., Wong-Foy A. G., Matzger A. J. (2009). J. Am. Chem. Soc..

[cit11] Farha O. K., Eryazici I., Jeong N. C., Hauser B. G., Wilmer C. E., Sarjeant A. A., Snurr R. Q., Nguyen S. T., Yazaydın A. Ö., Hupp J. T. (2012). J. Am. Chem. Soc..

[cit12] Cheng N., Ren L., Xu X., Du Y., Dou S. X. (2018). Adv. Energy Mater..

[cit13] Goetjen T. A., Liu J., Wu Y., Sui J., Zhang X., Hupp J. T., Farha O. K. (2020). Chem. Commun..

[cit14] Otake K., Cui Y., Buru C. T., Li Z., Hupp J. T., Farha O. K. (2018). J. Am. Chem. Soc..

[cit15] Carraro F., Chapman K., Chen Z., Dincă M., Easun T., Eddaoudi M., Farha O., Forgan R., Gagliardi L., Haase F., Harris D., Kitagawa S., Knichal J., Lamberti C., Lee J.-S. M., Leus K., Li J., Lin W., Lloyd G., Long J. R., Lu C., Ma S., McHugh L., Perez J. P. H., Ranocchiari M., Rosi N., Rosseinsky M., Ryder M. R., Ting V., van der Veen M., Voort P. V. D., Volkmer D., Walsh A., Woods D., Yaghi O. M. (2017). Faraday Discuss..

[cit16] Miner E. M., Wang L., Dincă M. (2018). Chem. Sci..

[cit17] Chang Z., Lin R.-B., Ye Y., Duan C., Chen B. (2019). J. Mater. Chem. A.

[cit18] Scott Bobbitt N., Mendonca M. L., Howarth A. J., Islamoglu T., Hupp J. T., Farha O. K., Snurr R. Q. (2017). Chem. Soc. Rev..

[cit19] Abánades Lázaro I., Forgan R. S. (2019). Coord. Chem. Rev..

[cit20] Veluswamy H. P., Kumar A., Seo Y., Lee J. D., Linga P. (2018). Appl. Energy.

[cit21] Lee J.-H., Siegelman R. L., Maserati L., Rangel T., Helms B. A., Long J. R., Neaton J. B. (2018). Chem. Sci..

[cit22] Wang L., Zheng M., Xie Z. (2018). J. Mater. Chem. B.

[cit23] Ibrahim M., Sabouni R., Husseini G. A. (2017). Curr. Med. Chem..

[cit24] Kotzabasaki M., Froudakis G. E. (2018). Inorg. Chem. Front..

[cit25] Li H., Lv N., Li X., Liu B., Feng J., Ren X., Guo T., Chen D., Stoddart J. F., Gref R., Zhang J. (2017). Nanoscale.

[cit26] Xie Y., Liu X., Ma X., Duan Y., Yao Y., Cai Q. (2018). ACS Appl. Mater. Interfaces.

[cit27] Teplensky M. H., Fantham M., Li P., Wang T. C., Mehta J. P., Young L. J., Moghadam P. Z., Hupp J. T., Farha O. K., Kaminski C. F., Fairen-Jimenez D. (2017). J. Am. Chem. Soc..

[cit28] Chen Y., Li P., Modica J. A., Drout R. J., Farha O. K. (2018). J. Am. Chem. Soc..

[cit29] Dolgopolova E. A., Rice A. M., Martin C. R., Shustova N. B. (2018). Chem. Soc. Rev..

[cit30] Wu Y.-P., Xu G.-W., Dong W.-W., Zhao J., Li D.-S., Zhang J., Bu X. (2017). Inorg. Chem..

[cit31] Zhao S.-S., Yang J., Liu Y.-Y., Ma J.-F. (2016). Inorg. Chem..

[cit32] Kobielska P. A., Howarth A. J., Farha O. K., Nayak S. (2018). Coord. Chem. Rev..

[cit33] Islamoglu T., Goswami S., Li Z., Howarth A. J., Farha O. K., Hupp J. T. (2017). Acc. Chem. Res..

[cit34] Deria P., Mondloch J. E., Karagiaridi O., Bury W., Hupp J. T., Farha O. K. (2014). Chem. Soc. Rev..

[cit35] Zhang H.-F., Li M., Wang X.-Z., Luo D., Zhao Y.-F., Zhou X.-P., Li D. (2018). J. Mater.
Chem. A.

[cit36] Garzón-Tovar L., Rodríguez-Hermida S., Imaz I., Maspoch D. (2017). J. Am. Chem. Soc..

[cit37] Cohen S. M. (2017). J. Am. Chem. Soc..

[cit38] Singh A., Karmakar S., Abraham I. M., Rambabu D., Dave D., Manjithaya R., Maji T. K. (2020). Inorg. Chem..

[cit39] Li J., Wang X., Zhao G., Chen C., Chai Z., Alsaedi A., Hayat T., Wang X. (2018). Chem. Soc. Rev..

[cit40] Dhaka S., Kumar R., Deep A., Kurade M. B., Ji S. W., Jeon B. H. (2019). Coord. Chem. Rev..

[cit41] Chen B., Yang Z., Zhu Y., Xia Y. (2014). J. Mater. Chem. A.

[cit42] Zhong G., Liu D., Zhang J. (2018). J. Mater. Chem. A.

[cit43] Wang T., Wang Y., Sun M., Hanif A., Wu H., Gu Q., Ok Y. S., Tsang D. C. W., Li J., Yu J., Shang J. (2020). Chem. Sci..

[cit44] Hu Z., Peng Y., Kang Z., Qian Y., Zhao D. (2015). Inorg. Chem..

[cit45] Zhang B., Luo Y., Kanyuck K., Saenz N., Reed K., Zavalij P., Mowery J., Bauchan G. (2018). RSC Adv..

[cit46] Denisov G. L., Primakov P. V., Korlyukov A. A., Novikov V. V., Nelyubina Yu. V. (2019). Russ. J. Coord. Chem..

[cit47] Shayegan H., Ali G. A. M., Safarifard V. (2020). ChemistrySelect.

[cit48] Fu Z., Guo W., Dang Z., Hu Q., Wu F., Feng C., Zhao X., Meng W., Xing B., Giesy J. P. (2017). Environ. Sci. Technol..

[cit49] Liang W., Li M., Zhang Z., Jiang Y., Awasthi M. K., Jiang S., Li R. (2018). Int. J. Biol. Macromol..

[cit50] Zhu Y., Zheng Y., Wang W., Wang A. (2015). J. Water Process Eng..

[cit51] Borji H., Ayoub G. M., Bilbeisi R., Nassar N., Malaeb L. (2020). Water. Air. Soil Pollut..

[cit52] Bolisetty S., Peydayesh M., Mezzenga R. (2019). Chem. Soc. Rev..

[cit53] Xia M., Chen Z., Li Y., Li C., Ahmad N. M., Cheema W. A., Zhu S. (2019). RSC Adv..

[cit54] Kumar P., Pournara A., Kim K.-H., Bansal V., Rapti S., Manos M. J. (2017). Prog. Mater. Sci..

[cit55] Tunsu C., Wickman B. (2018). Nat. Commun..

[cit56] Bengtsson M. K. O., Tunsu C., Wickman B. (2019). Ind. Eng. Chem. Res..

[cit57] Li J., Liu Y., Ai Y., Alsaedi A., Hayat T., Wang X. (2018). Chem. Eng. J..

[cit58] Abbas K., Znad H., Awual Md. R. (2018). Chem. Eng. J..

[cit59] Samyn P., Barhoum A., Öhlund T., Dufresne A. (2018). J. Mater. Sci..

[cit60] Oehmen A., Vergel D., Fradinho J., Reis M. A. M., Crespo J. G., Velizarov S. (2014). J. Hazard. Mater..

[cit61] Yee K.-K., Reimer N., Liu J., Cheng S.-Y., Yiu S.-M., Weber J., Stock N., Xu Z. (2013). J. Am. Chem. Soc..

[cit62] Ke F., Qiu L.-G., Yuan Y.-P., Peng F.-M., Jiang X., Xie A.-J., Shen Y.-H., Zhu J.-F. (2011). J. Hazard. Mater..

[cit63] He J., Yee K.-K., Xu Z., Zeller M., Hunter A. D., Chui S. S.-Y., Che C.-M. (2011). Chem. Mater..

[cit64] Bhattacharjee S., Lee Y.-R., Ahn W.-S. (2015). CrystEngComm.

[cit65] Saleem H., Rafique U., Davies R. P. (2016). Microporous Mesoporous Mater..

[cit66] Liang L., Chen Q., Jiang F., Yuan D., Qian J., Lv G., Xue H., Liu L., Jiang H.-L., Hong M. (2016). J. Mater. Chem. A.

[cit67] Yang P., Shu Y., Zhuang Q., Li Y., Gu J. (2019). Chem. Commun..

[cit68] Liu F., Xiong W., Feng X., Shi L., Chen D., Zhang Y. (2019). J. Hazard. Mater..

[cit69] Jian M., Liu B., Liu R., Qu J., Wang H., Zhang X. (2015). RSC Adv..

[cit70] Jaafar A., Fix-Tailler A., Mansour N., Allain M., Shebaby W. N., Faour W. H., Tokajian S., El-Ghayoury A., Naoufal D., Bouchara J.-P., Larcher G., Ibrahim G. (2020). Appl. Organomet. Chem..

[cit71] Andres S. A., Bajaj K., Vishnosky N. S., Peterson M. A., Mashuta M. S., Buchanan R. M., Bates P. J., Grapperhaus C. A. (2020). Inorg. Chem..

[cit72] Ibrahim A. B. M., Farh M. K., Mayer P. (2018). Inorg. Chem. Commun..

[cit73] Ramachandran E., Gandin V., Bertani R., Sgarbossa P., Natarajan K., Bhuvanesh N. S. P., Venzo A., Zoleo A., Mozzon M., Dolmella A., Albinati A., Castellano C., Reis Conceição N., Guedes da Silva M. F. C., Marzano C. (2020). Molecules.

[cit74] Sankaraperumal A., Shetty A. N., Karthikeyan J. (2015). Synth. React. Inorg., Met.-Org., Nano-Met. Chem..

[cit75] Chu J., Ke F.-S., Wang Y., Feng X., Chen W., Ai X., Yang H., Cao Y. (2020). Commun. Chem..

[cit76] Kim M., Cahill J. F., Fei H., Prather K. A., Cohen S. M. (2012). J. Am. Chem. Soc..

[cit77] Becke A. D. (1993). J. Chem. Phys..

[cit78] Frisch M. J. , Trucks G. W., Schlegel H. B., Scuseria G. E., Robb M. A., Cheeseman J. R., Scalmani G., Barone V., Petersson G. A., Nakatsuji H., Li X., Caricato M., Marenich A., Bloino J., Janesko B. G., Gomperts R., Mennucci B., Hratchian H. P., Ortiz J. V., Izmaylov A. F., Sonnenberg J. L., Williams-Young D., Ding F., Lipparini F., Egidi F., Goings J., Peng B., Petrone A., Henderson T., Ranasinghe D., Zakrzewski V. G., Gao J., Rega N., Zheng G., Liang W., Hada M., Ehara M., Toyota K., Fukuda R., Hasegawa J., Ishida M., Nakajima T., Honda Y., Kitao O., Nakai H., Vreven T., Throssell K., Montgomery Jr J. A., Peralta J. E., Ogliaro F., Bearpark M., Heyd J. J., Brothers E., Kudin K. N., Staroverov V. N., Keith T., Kobayashi R., Normand J., Raghavachari K., Rendell A., Burant J. C., Iyengar S. S., Tomasi J., Cossi M., Millam J. M., Klene M., Adamo C., Cammi R., Ochterski J. W., Martin R. L., Morokuma K., Farkas O., Foresman J. B., and Fox D. J., Gaussian 09, Revision E.01, Gaussian, Inc., Wallingford CT, 2016

[cit79] Lee C., Yang W., Parr R. G. (1988). Phys. Rev. B.

[cit80] Phan A., Doonan C. J., Uribe-Romo F. J., Knobler C. B., O'Keeffe M., Yaghi O. M. (2010). Acc. Chem. Res..

[cit81] Mahmoud M. E., Amira M. F., Seleim S. M., Mohamed A. K. (2017). J. Chem. Eng. Data.

[cit82] Duwiejuah A. B., Cobbina S. J., Quainoo A. K., Abubakari A. H., Bakobie N. (2018). J. Health Pollut..

[cit83] Adelaja O. A., Bankole A. C., Oladipo M. E., Lene D. B. (2019). Int. J. Energy Water Resour..

