# Supplementary material for: Thiosemicarbazone modified zeolitic imidazolate framework (TSC-ZIF) for mercury(ii) removal from water
Source: RSC Adv. 2021 Apr 30;11(27):16192–9. doi: 10.1039/d1ra02025k (PMC9030954; doi:10.1039/d1ra02025k)
Supplement: RA-011-D1RA02025K-s001 [file RA-011-D1RA02025K-s001.pdf]

Electronic Supplementary Material (ESI) for RSC Advances.
This journal is © The Royal Society of Chemistry 2021

Supporting Information

Thiosemicarbazone Modified Zeolitic Imidazolate Framework (TSC-ZIF) for Mercury(II) Removal from Water

Amani Jaafar,[a] Carlos Platas-Iglesias,[b] and Rana A. Bilbeisi *[a]

Table of Contents

1. General **2**

2. Synthesis and characterization of the ZIF-8, Ald-ZIF and TSC-ZIF **4**

2.1. Hydrothermal procedure for the preparation of ZIF-8 .... **4**

2.2. Preparation of the Ald-ZIF **4**

2.2.1. Hydrothermal procedure for the preparation of **MIM_{15}:AldIM$_1$:Zn** 4

2.2.2. Solvothermal procedure for $MIM_{3.5}$:AldIM$_1$:Zn **4**

2.3. Preparation of TSC-ZIF by dynamic post-synthetic modification of the Ald-ZIF **5**

2.3.1. Preparation of **MIM_{15}:Bisthio$_1$:Zn** 5

2.3.2 Preparation of **MIM_{15}:Thio$_1$:Zn** 5

2.3.3. Preparation of **$MIM_{3.5}$:Bisthio$_1$:Zn** 5

2.3.4. Preparation of **$MIM_{3.5}$:Thio$_1$:Zn** 5

3. Hg^{2+} removal efficiency from water **17**

3.1. Kinetic experiments **17**

3.1.1. Percentage removal 18

3.2. Hg^{2+} ions sorption isotherm **19**

3.2.1. Adsorption isotherm models 19

3.2.2. Adsorption Kinetic 21

3.3. Ion selectivity tests **25**

3.3.1. Competitive binding: binary system (Hg^{2+} and Pb^{2+}) 25

3.3.2. Competitive binding: tertiary system (Hg^{2+}, Pb^{2+} and Cd^{2+}) 26

4. Regeneration of $MIM_{3.5}$:Thio$_1$:Zn **26**

Figure S1: FT-IR spectra of **ZIF-8, Ald-ZIF** and **TSC-ZIF** 6
Figure S2: ^{1}H NMR for the digested **ZIF-8** (500 MHz, D_2O-d_2 and CD_3COOD-d_4) 7
Figure S3: ^{13}C NMR for the digested **ZIF-8** (125 MHz, D_2O-d_2 and D_3COOD-d_4)....7
Figure S4: ^{1}H NMR of the digested **MIM_{15}:AldIM$_1$:Zn** (500 MHz, DMSO-d_6 and CD_3COOD-d_4) .8
Figure S5: ^{1}H NMR of the digested **MIM_{15}:Bisthio$_1$:Zn** (500 MHz, DMSO-d_6 and CD_3COOD-d_4) 9
Figure S6: ^{1}H NMR of the digested **MIM_{15}:Thio$_1$:Zn** (500 MHz, DMSO-d_6 and CD_3COOD-d_4)..10
Figure S7: ^{1}H NMR of the digested **$MIM_{3.5}$:AldIM$_1$:Zn** (500 MHz, D_2O-d_2 and CD_3COOD-d_4) ...11
Figure S8: ^{13}C NMR of the digested **$MIM_{3.5}$:AldIM$_1$:Zn** (125 MHz, D_2O-d_2 and CD_3COOD-d_4)..12
Figure S9: ^{13}C NMR of the digested **$MIM_{3.5}$:Bisthio$_1$:Zn** (500 MHz, D_2O-d_2 and CD_3COOD-d_4) 12
Figure S10: ^{1}H NMR of the digested **$MIM_{3.5}$:Thio$_1$:Zn** (500 MHz, D_2O-d_2 and CD_3COOD-d_4) ...13
Figure S11: ^{13}C NMR of the digested **$MIM_{3.5}$:Thio$_1$:Zn** (125 MHz, D_2O-d_2 and CD_3COOD-d_4)..14
Figure S12: PXRD pattern of simulated **ZIF-8**....14
Figure S13: N_2 adsorption (filled circles) and desorption (empty circles) isotherms at 77 K of **ZIF-8**, **Ald-ZIF** and **TSC-ZIF**....15
Figure S14: EDX spectra of SEM images for (a) **$MIM_{3.5}$:AldIM$_1$:Zn**, (b) **$MIM_{3.5}$:Bisthio$_1$:Zn** and (c) **$MIM_{3.5}$:Thio$_1$:Zn**....16
Figure S15: Thermogravimetric analysis of **ZIF-8**, **Ald-ZIF** and **TSC-ZIF** 17
Figure S16: Pictures of **$MIM_{3.5}$:Thio:Zn (originally yellow in colour)** solutions with different concentrations of Hg^{2+} solutions (colourless) The gray-black colour of the **$MIM_{3.5}$:Thio$_1$:Zn**. indicates the adsorption of Hg^{2+}. 18
Figure S17: Percentage removal of Hg^{2+} with time using **ZIF-8**, **Ald-ZIF** and **TSC-ZIF,** ($[Hg^{2+}]$ =100mg/L)....19
Figure S18: Langmuir isotherm plots for: (a) **ZIF-8** and **MIM_{15}:AldIM$_1$:Zn**, (b) **MIM_{15}:Bisthio$_1$:Zn**, and **MIM_{15}:Thio$_1$:Zn** 21
Figure S19: The pseudo-second order kinetic plot for the adsorption ($[Hg^{2+}]_i$ = 100 ppm) for: (a) **ZIF-8**, (b) **MIM_{15}:Ald$_1$:Zn**, (c) **MIM_{15}:Bithio$_1$:Zn** and (d) **MIM_{15}:Thio$_1$:Zn** 22
Figure S20: The pseudo-second order kinetic plots for the adsorption ($[Hg^{2+}]$=400 ppm) for : (a) **$MIM_{3.5}$:Ald$_1$:Zn**, (b) **$MIM_{3.5}$:Bithio$_1$:Zn** and (c) **$MIM_{3.5}$:Thio$_1$:Zn** 23
Figure S21: SEM images for **$MIM_{3.5}$:Thio$_1$:Zn** after adsorption of Hg^{2+} 24
Figure S22: EDX spectra of SEM images for the **$MIM_{3.5}$:Thio$_1$:Zn** after the adsorption of Hg^{2+} 24
Figure S23: TGA profile of the **$MIM_{3.5}$:Thio$_1$:Zn** before and after adsorption of Hg^{2+} 25

Table S1: Freundlich equation parameters for Hg^{2+} ions adsorption onto the as-prepared **ZIF-8**, **Ald-ZIF** and **TSC-ZIF** 21
Table S2: Pseudo-second order equation parameters for Hg^{2+} ions adsorption onto the as-prepared **ZIF-8**, **Ald-ZIF** and **TSC-ZIF**....23

1. General

1.1. Chemicals and materials

All chemicals and reagents used in this study were of analytical grade, obtained from commercial suppliers, and used without further purification unless otherwise noted. Imidazole-4-carbaldehyde (97%) and 2-methylimidazole (99%) was purchased from Acros. Zinc (II) nitrate hexahydrate and zinc (II) acetate dihydrate were purchased from fisher scientific. Thiocarbohydrazide (98%), thiosemicarbazide, Mercury(II) trifluoroacetate (98%), Lead nitrate dihydrated, and Cadmium nitrate dehydrated were purchased from Aldrich.

1.2. Instruments and methods

All synthesized samples were fully characterized using FTIR, NMR, PXRD, N_2 sorption, SEM, and TGA. **ATR-FTIR** spectra (4 scans, 4 cm^{-1} resolution, wavenumber range 4000 – 650 cm^{-1}) were obtained using a Perkin Elmer FTIR Spectrum 2000 Spectrophotometer with a diamond/ZnSe crystal window. All spectra were recorded under ambient conditions. SPECTRUM software (Perkin Elmer) was used for data collection. The IR bands are reported in wavenumbers (cm^{-1}).

Nuclear Magnetic Resonance (**NMR**) spectra were acquired after the digestion of MOFs by 20% deuterated acetic acid on an AC500 Bruker spectrometer (^1H and ^{13}C NMR at 500 MHz). Chemical shifts were recorded in delta (δ) units and expressed as ppm values relative to the internal standard TMS.

Powder X-ray diffraction (**PXRD**) patterns of the prepared MOFs were recorded using a Bruker D8 advance X-ray diffractometer (Bruker AXS GmbH, Karlsruhe, Germany, working at 40 kV and current 40 mA, 2θ range: 5–50°, increment: 0.02°) using Cu Kα radiation (k=1.5418 Å).

Before N_2 sorption measurements, samples were degassed to remove adsorbed water and other solvents using vacuum and flow degassing. Vacuum degassing was performed at 150 °C for 12 h, followed by degassing under a flow of nitrogen at 150 °C overnight in a BET cell. After degassing, the cell was then placed in the measurement unit of Micro metrics Gemini VII 2390p surface area analyzer (**BET**), and the N_2 sorption was performed at 77 K.

Scanning electron microscopy and EDX (**SEM-EDX**) imaging was performed using a MIRA3 Tescan electron microscope after the samples were coated with a thin layer (20 nm) of platinum.

Thermal stabilities of the prepared MOFs were measured using thermogravimetric analysis (**TGA**), which was performed using a Netzsch TG 209 F1 Libra apparatus. Samples were heated from 30 to 1000 °C at a heating rate of 10 K min^{-1}.

DFT calculations were performed using the Gaussian09 program package (revision E.01),[1] employing the hybrid b3lyp functional [2] and the standard 6-31G(d,p) basis set. Default integration grids and scf convergence thresholds were used.

The concentration of mercury(II) was determined by an atomic absorption spectrometer (**AAS**), iCE 3000 series, with a deuterium background corrector equipped with a single element hollow

cathode lamp that was used in the study. All measurements were carried out in an air/acetylene flame

2. Synthesis and characterization of the ZIF-8, Ald-ZIF and TSC-ZIF

2.1. Hydrothermal procedure for the preparation of ZIF-8

ZIF-8 was prepared through the addition of a solution of $Zn(OAC)_2.2H_2O$ (540 mg, 2.4 mmol, 2 eq) in 50 ml deionized water to a solution of 2-methylimidazole (MIM) (1.6 g, 19.7 mmol, 16 eq) in 50 ml methanol. The mixture was stirred at room temperature for 60 min. The obtained crystals were washed several times with methanol and dried at 40 ^oC for 24 h (white fine crystals, Yield: 93%). IR (cm^{-1}): v~ = 1614 (C=N); ^1H NMR (500 MHz, 80 % D_2O-d_2 and 20 % CD_3COOD-d_4): δ= 2.04 (s, 3H, $-CH_3)_{MIM}$, 6.73 (s, $2H)_{MIM}$; ^{13}C NMR (125 MHz, 80 % D_2O-d_2 and 20 % CD_3COOD-d_4): 143 $(C=N)_{MIM}$, 9.86 $(CH_3)_{MIM}$, 117.73 ppm (Cycl $imidazole)_{MIM}$.

2.2. Preparation of the Ald-ZIF

2.2.1. Hydrothermal procedure for the preparation of **MIM_{15}:$AldIM_1$:Zn**

MIM_{15}:$AldIM_1$:Zn was prepared through the addition of a solution of $Zn(OAC)_2.2H_2O$ (540 mg, 2.4 mmol, 2 eq) in 50 ml deionized water to a solution of 2-methylimidazole (MIM) eq) (1.5 g, 18.2 mmol, 15 eq) and carbaldehyde-4-imidazole (AldIM) (116 mg,1.21 mmol, 1 eq) in 50 mL methanol. The mixture was stirred at room temperature for 60 min.The obtained crystals were washed several times with methanol and dried at 40 ^oC for 24 h (Off-white Fine crystals, Yield: 75%). IR (cm^{-1}): v~ = 1614 (C=N), 1580 (C=O); ^1H NMR (500 MHz, 80 % DMSO-d_6 and 20 % CD_3COOD-d_4): δ= 2.37 (s, 15*3H, $-CH_3)_{MIM}$, 7.08 (s, 15* $2H)_{MIM}$, 7.93 (s, $1*H)_{AldIM}$, 7.98(s,1* $H)_{AldIM}$, 7.96 (s, 1* $H)_{AldIM}$.

2.2.2. Solvothermal procedure for **$MIM_{3.5}$:$AldIM_1$:Zn**

The **$MIM_{3.5}$:$AldIM_1$:Zn** was obtained by heating a solution of $Zn(NO_3)_2.6H_2O$ (298 mg, 1 mmol, 2.25 eq), 2-methylimidazole (MIM) (150 mg, 1.82 mmol, 3.5 eq) and carbaldehyde-4-imidazole (AldIM) (50 mg, 0.52 mmol,1 eq) in *N*, *N*-dimethylformamide (DMF, 4 mL) for 72 h at 110 ^oC in sand bath at an oven.The obtained crystals were washed several times with methanol and dried at 70 ^oC for 24 h (Crystals, yellow/brown, Yield: 54%). IR (cm^{-1}): v~ = 1614 (C=N), 1580 (C=O); ^1H NMR (500 MHz, 80 % D_2O-d_2 and 20 % CD_3COOD-d_4): δ= 2.03 (s, 3.5*3H, $-CH_3)_{MIM}$, 6.72 (s, 3.5* $2H)_{MIM}$, 7.60 (s, $1*H)_{AldIM}$, 7.77(s,1* $H)_{AldIM}$, 9.17 (s, 1* $H)_{AldIM}$. ^{13}C NMR (125 MHz, 80 % D_2O-d_2 and 20 % CD_3COOD-d_4): 143.73 $(C=N)_{MIM}$, 8.24 $(CH_3)_{MIM}$, 117.73 (Cyl $imidazole)_{MIM}$, 139.23, 134.84, 129.71 (Cycl $Imidazole)_{AldIM}$, 183 ppm $(C=O)_{AldIM}$.

2.3. Preparation of TSC-ZIF by dynamic post-synthetic modification of the Ald-ZIF

2.3.1. Preparation of **MIM_{15}:$Bisthio_1$:Zn**

Vacuum dried crystals of **MIM_{15}:$AldIM_1$:Zn**, 200 mg (containing 0.138 mmol AldIM, 1 eq) were suspended in 3 mL deionized water with bisthiosemicarbazone (14.6 mg, 0.138 mmol, 1 eq) and refluxed for 24 h. The reaction mixture was filtered, and the solid was washed 2-3 times with fresh methanol (10 mL). The solid was dried under vacuum at 40 ^oC for 24 h (fine crystals, Beige yield: 40%). IR (cm^{-1}): v~ = 1614 (C=N), 1236, 842 (C=S+C=N, C=S), 2122 (NH-C=S), 3250 ($NH-NH_2$) *cm^{-1}*; ^1H NMR (500 MHz, 80 % DMSO-d_6 and 20 % $CD_3COOD-d_4$): δ= 2.40 (s, 15*3H, $-CH_3)_{MIM}$, 7.17 (s, 15* $2H)_{MIM}$, 7.92 (s, $1*H_{CH=N})_{Bisthio}$, 7.88(s,1* $H)_{Bisthio}$, 7.90 (s, 1* $H)_{Bisthio}$, 7.56 (s, 1* $H)_{Bisthio}$, 7.46 (s, 1* $H)_{Bisthio}$, 7.39 (s, 1* $H)_{Bisthio.}$

2.3.2 Preparation of **MIM_{15}:$Thio_1$:Zn**

Vacuum dried color crystals of **MIM_{15}:$AldIM_1$:Zn**, 200 mg (containing 0.138 mmol AldIM, 1 eq) was suspended in 3 mL deionized water with thiosemicarbazide (12.5 mg, 0.138 mmol, 1 eq) and refluxed for 24 h. The reaction mixture was filtered, and the solid was washed three times with fresh methanol (10 mL). The solid was dried under vacuum at 40 ^oC for 24 h (light yellow fine crystals, Yield: 77%). IR (cm^{-1}): v~ = 1614 (C=N), 1236, 842 (C=S+C=N, C=S), 2122 cm^{-1} (NH-C=S); ^1H NMR (500 MHz, 80 % DMSO-d_6 and 20 % $CD_3COOD-d_4$): δ= 2.34 (s, 15*3H, $-CH_3)_{MIM}$, 7.07 (s, 15* $2H)_{MIM}$, 7.93 (s, $1*H_{CH=N})_{Thio}$, 7.70(s,1* $H)_{Thio}$, 7.58 (s, 1* $H)_{Thio}$, 7.48 (s, 1* $H)_{Thio}$, 8.17 (s, 1* $H)_{Thio}$

2.3.3. Preparation of **$MIM_{3.5}$:$Bisthio_1$:Zn**

Vacuum dried crystals **$MIM_{3.5}$:$AldIM_1$:Zn** on a vacuum oven, 200 mg (containing 0.59 mmol AldIM, 1eq) were suspended in 5 mL deionized water with bisthiosemicarbazone (63 mg, 0.59 mmol, 1 eq) and refluxed for 24 h. The reaction mixture was filtered, and the solid was washed 2-3 times with fresh methanol (10 mL). The solid was dried under vacuum at 40 ^oC for 24 h (fine crystals, white-yellow, yield: 57%). IR (cm^{-1}): v~ = 1614 (C=N), 1236, 842 (C=S+C=N, C=S), 2122 (NH-C=S), 3250 ($NH-NH_2$) cm^{-1}; ^{13}C NMR (125 MHz, 80 % D_2O-d_2 and 20 % $CD_3COOD-d_4$): 143.76 $(C=N)_{MIM}$, 9.17 $(CH_3)_{MIM}$, 117.75 (Cyl $imidazole)_{MIM}$, 122.78, 121.78, 142.03.71 (Cycl $Imidazole)_{Bithio}$, 136.17 $(C=N)_{Bithio}$, 178.07 ppm $(C=S)_{Bisthio}$.

2.3.4. Preparation of **$MIM_{3.5}$:$Thio_1$:Zn**

Vacuum dried crystals **$MIM_{3.5}$:$AldIM_1$:Zn**, 200 mg (containing 0.59 mmol AldIM, 1 eq) were suspended in 3 mL deionized water with thiosemicarbazide (54 mg, 0.59 mmol, 1eq) and refluxed for 24 h. The reaction mixture was filtered, and the solid was washed 2-3 times with fresh methanol (10 mL). The solid was dried under vacuum at 40 ^oC for 24 h (light yellow fine crystals,

Yield: 62%). IR (cm^{-1}): v~ = 1614 (C=N), 1236, 842 (C=S+C=N, C=S), 2122 cm^{-1}(NH-C=S). ^{1}H NMR (500 MHz, 80 % D_2O-d_2 and 20 % CD_3COOD-d_4): δ= 2.40 (s, 3.5*3H, -CH_3)$_{MIM}$, 7.15 (s, 3.5* 2H)$_{MIM}$, 7.95(s, 1*H)$_{Thio}$, 7.36 (s, 2* H)$_{Thio}$, 8.04 (s, 2* H)$_{Thio}$, 8.08 (s, 1* H)$_{Thio}$; ^{13}C NMR (125 MHz, 80 % D_2O-d_2 and 20 % CD_3COOD-d_4): 143.76 (C=N)$_{MIM}$, 9.17 (CH_3)$_{MIM}$, 117.75 (Cyl imidazole)$_{MIM}$, 122.78, 121.78, 142.03.71 (Cycl Imidazole)$_{Thio}$, 136.17 (C=N)$_{Thio}$, 178.07 ppm (C=S)$_{Thio}$.

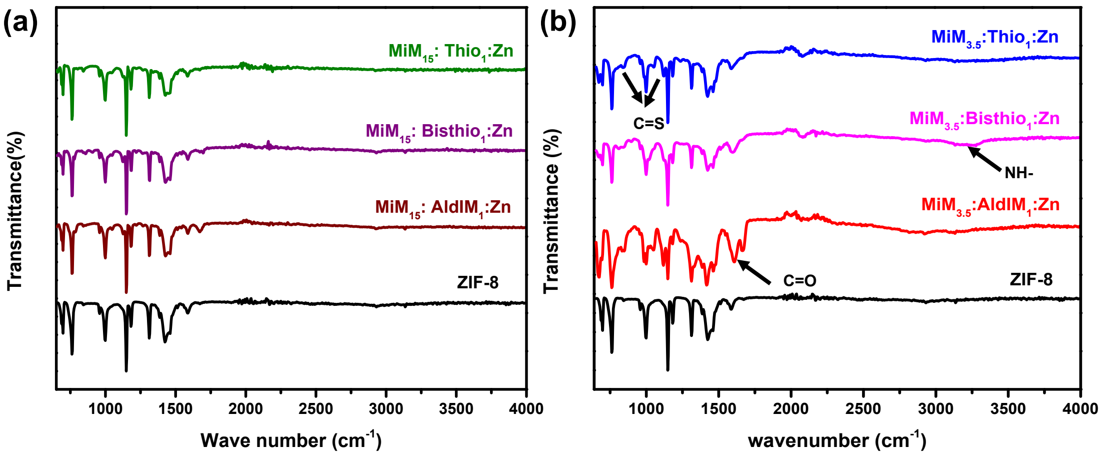

Figure S1: FT-IR spectra of **ZIF-8, Ald-ZIF** and **TSC-ZIF**

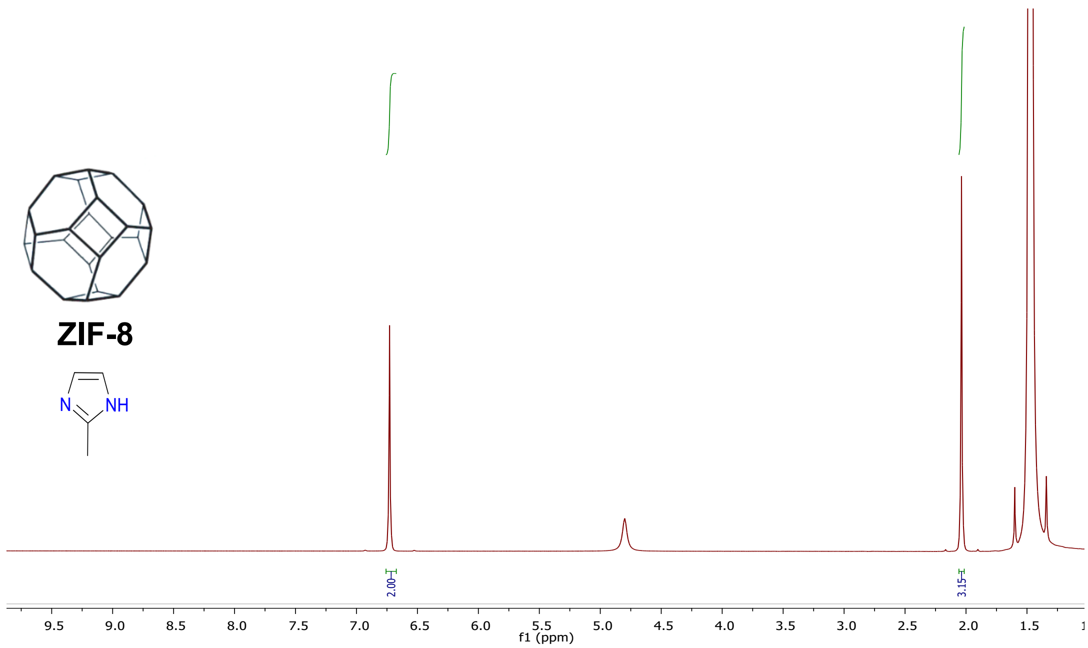

Figure S2: *^{1}H NMR for the digested **ZIF-8** (500 MHz, D_2O-d_2 and CD_3COOD-d_4)*

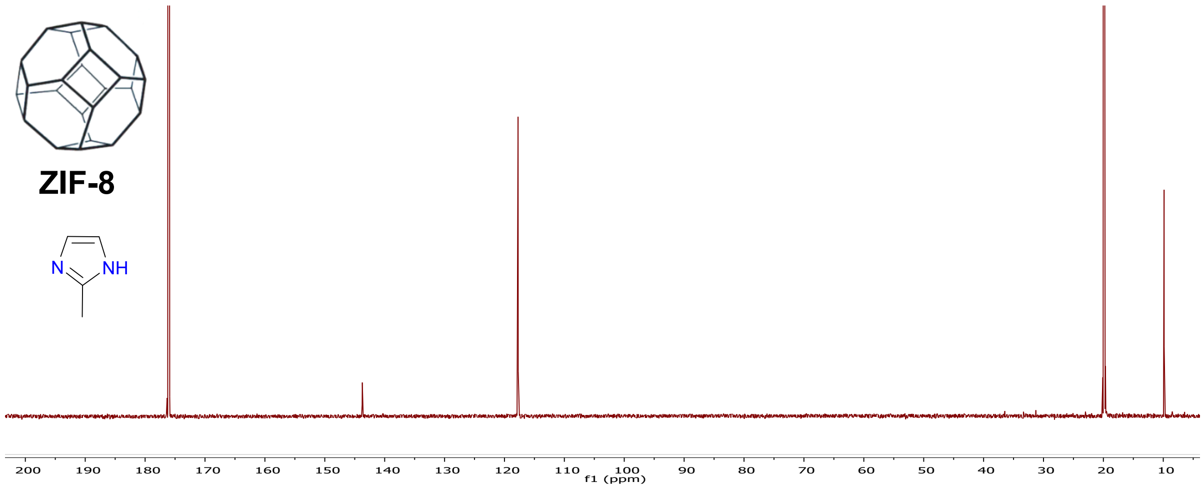

Figure S3: *^{13}C NMR for the digested **ZIF-8** (125 MHz, D_2O-d_2 and D_3COOD-d_4)*

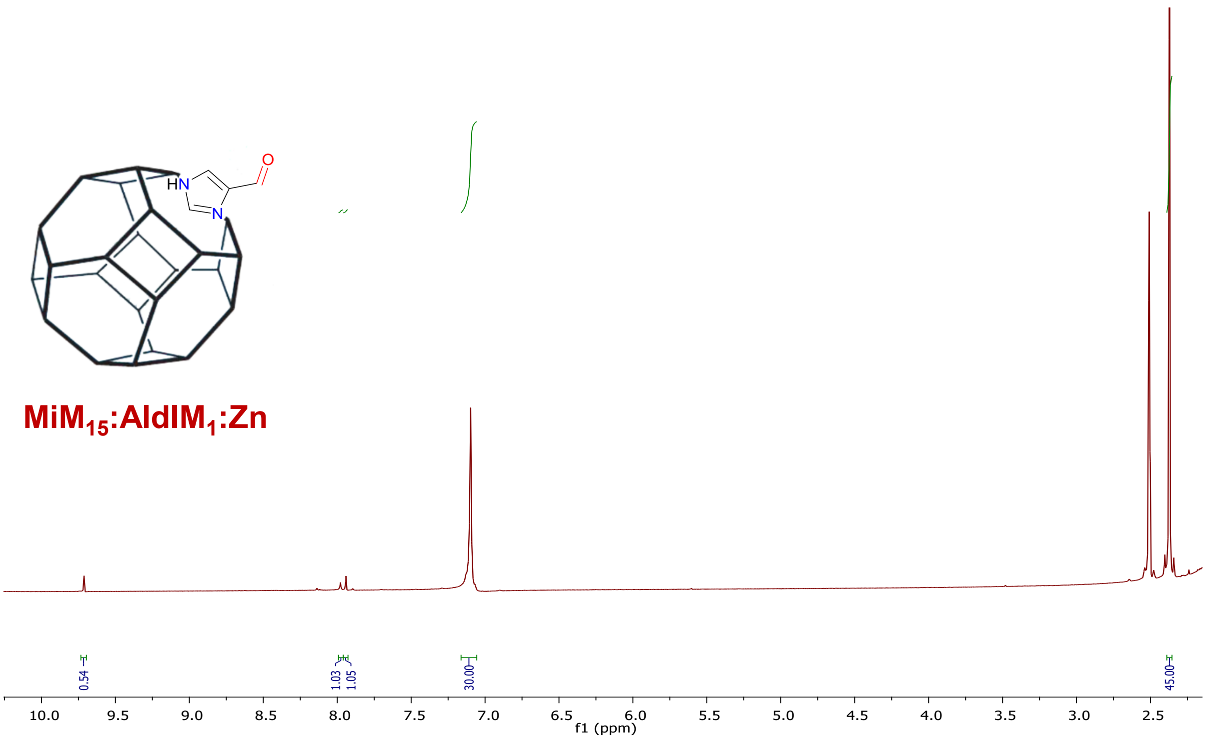

Figure S4: ^{1}H NMR of the digested **MIM_{15}:$AldIM_1$:Zn** (500 MHz, DMSO-d_6 and CD_3COOD-d_4)

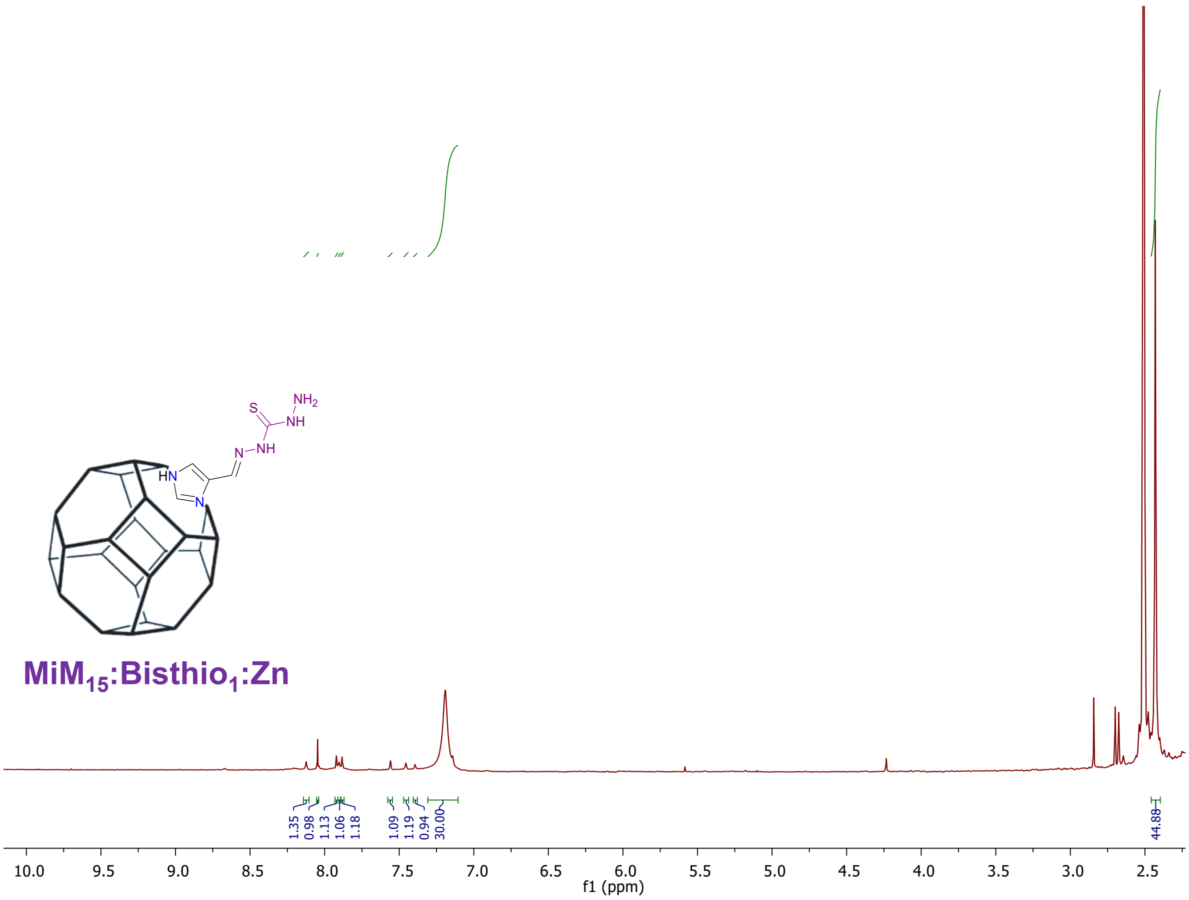

Figure S5: ^{1}H NMR of the digested **MIM$_{15}$:Bisthio$_{1}$:Zn** (500 MHz, DMSO-d_6 and CD_3COOD-d_4)

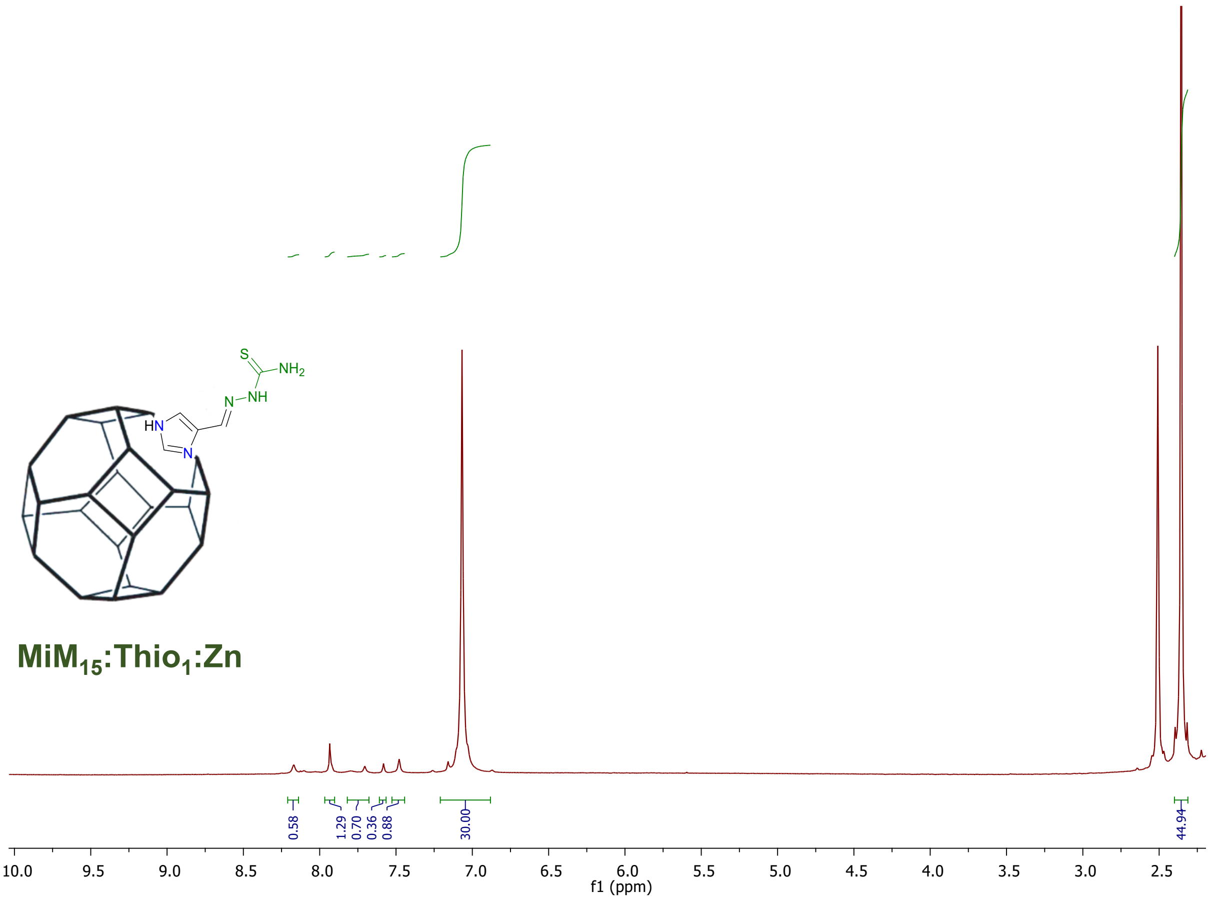

Figure S6: ^{1}H NMR of the digested **MIM$_{15}$:Thio$_1$:Zn** (500 MHz, DMSO-d$_6$ and CD_3COOD-d$_4$)

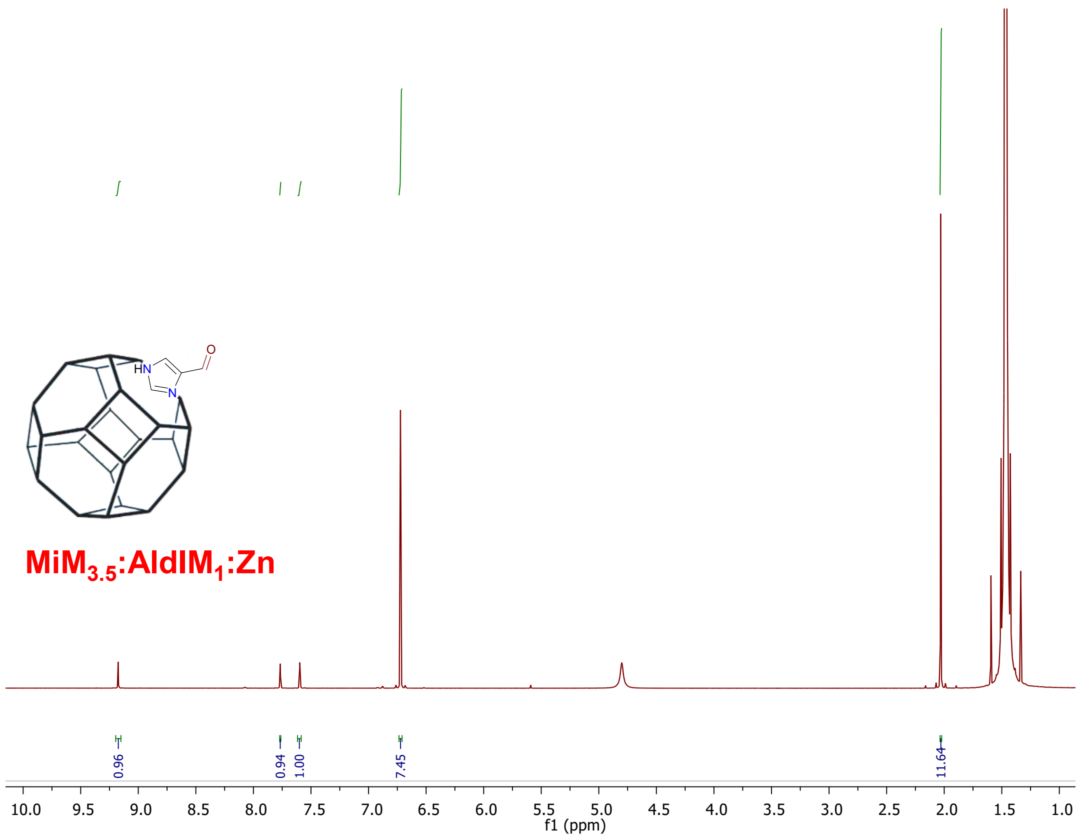

Figure S7: ^{1}H NMR of the digested **MIM$_{3.5}$:AldIM$_1$:Zn** (500 MHz, D_2O-d$_2$ and CD_3COOD-d$_4$)

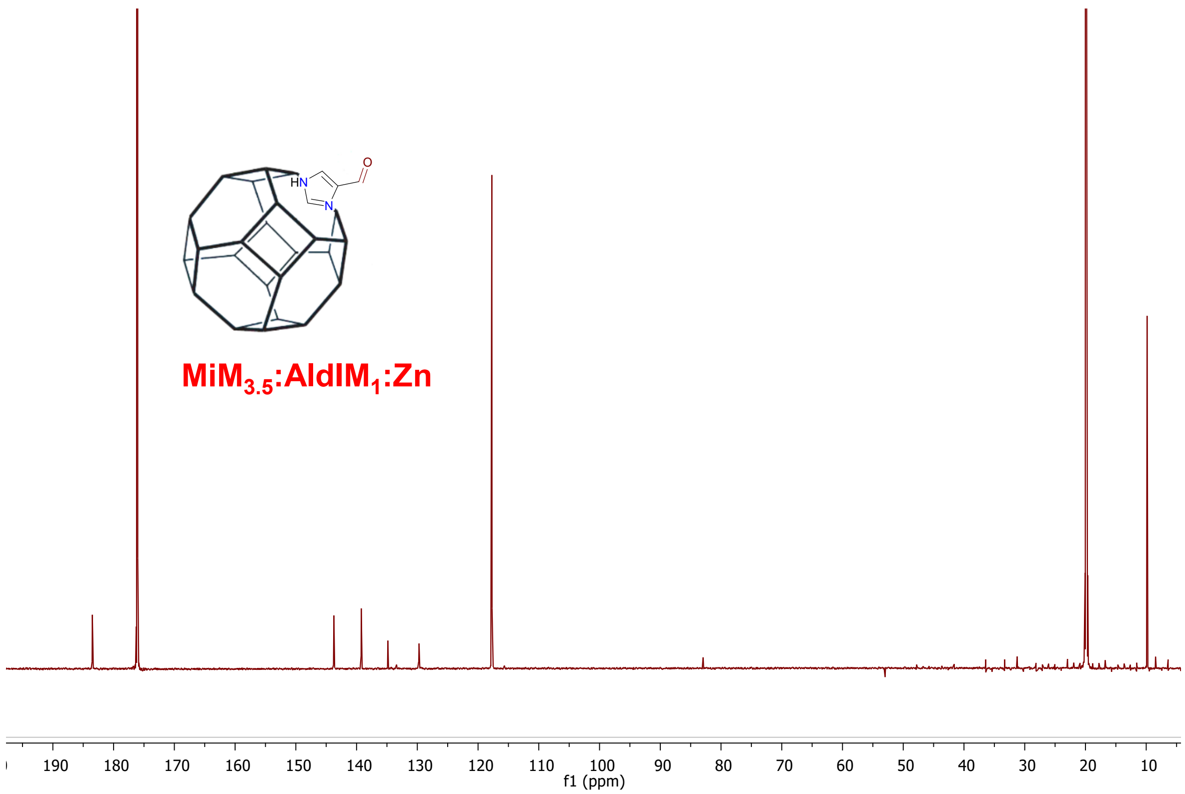

Figure S8: ^{13}C NMR of the digested **MIM$_{3.5}$:AldIM$_1$:Zn** (125 MHz, D_2O-d_2 and CD_3COOD-d_4)

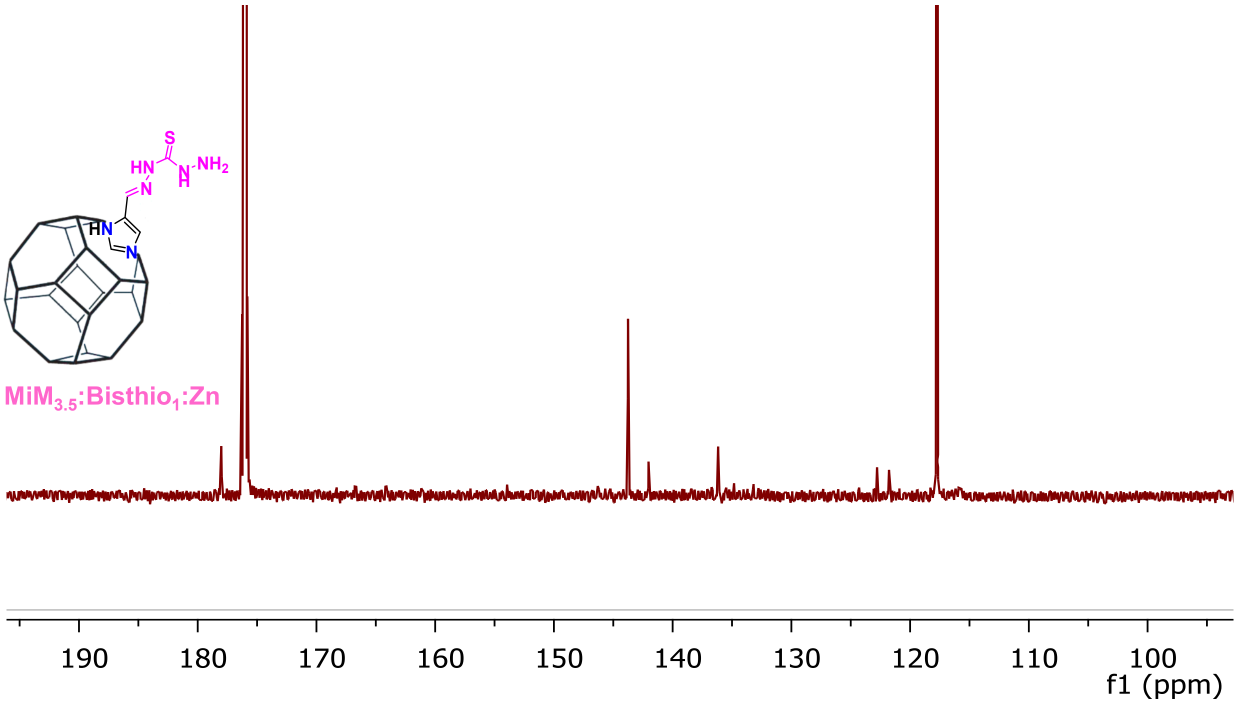

Figure S9: ^{13}C NMR of the digested **MIM$_{3.5}$:Bisthio$_1$:Zn** (500 MHz, D_2O-d_2 and CD_3COOD-d_4)

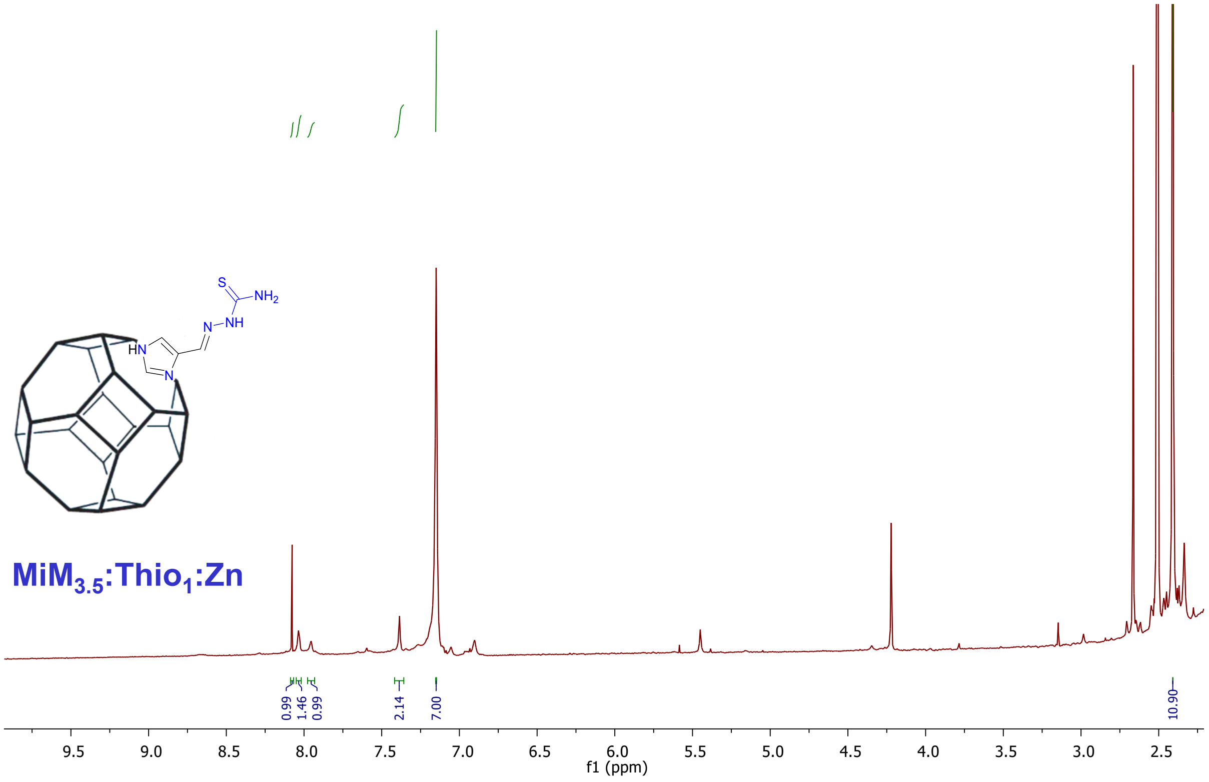

Figure S10: ^{1}H NMR of the digested **MIM$_{3.5}$:Thio$_1$:Zn** (500 MHz, D_2O-d_2 and CD_3COOD-d_4)

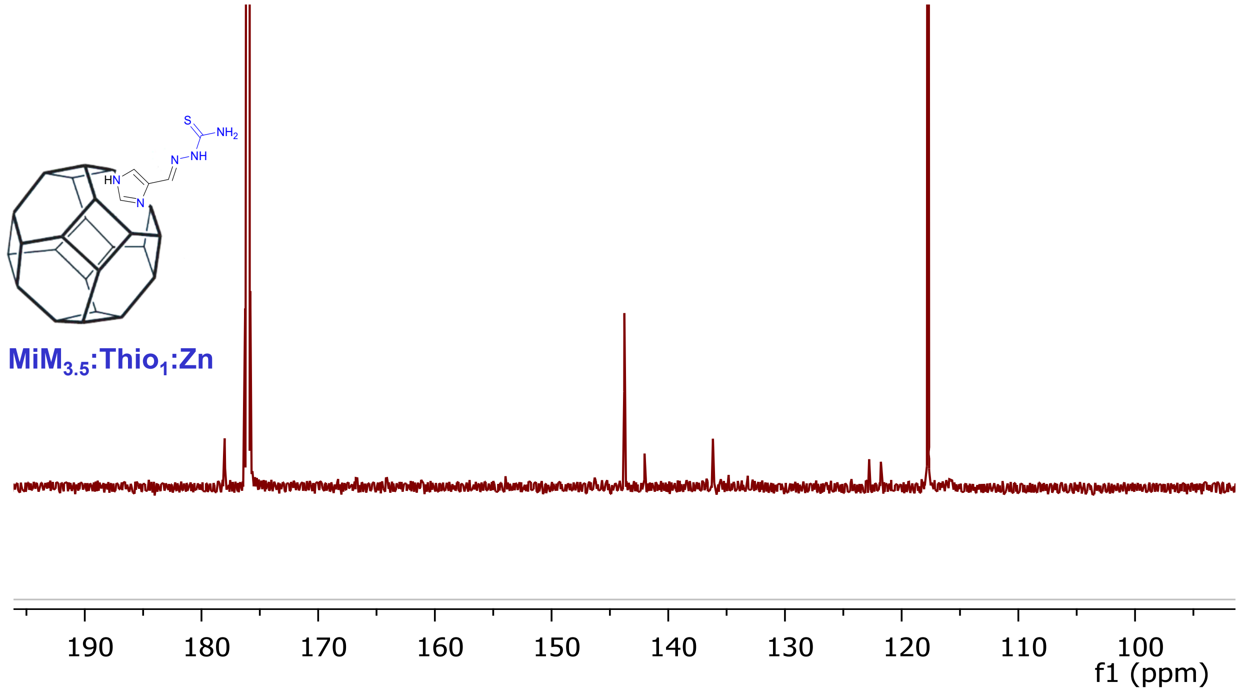

Figure S11: ^{13}C NMR of the digested **$MIM_{3.5}$:$Thio_1$:Zn** (125 MHz, D_2O-d_2 and CD_3COOD-d_4)

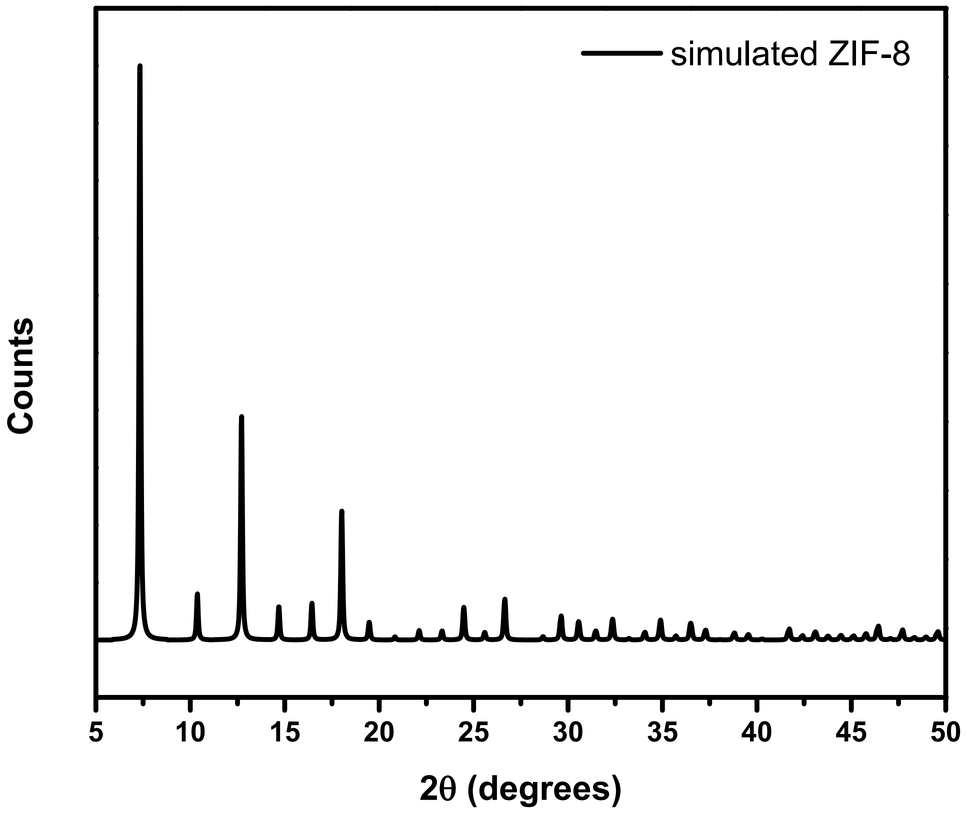

Figure S12: PXRD pattern of simulated **ZIF-8**

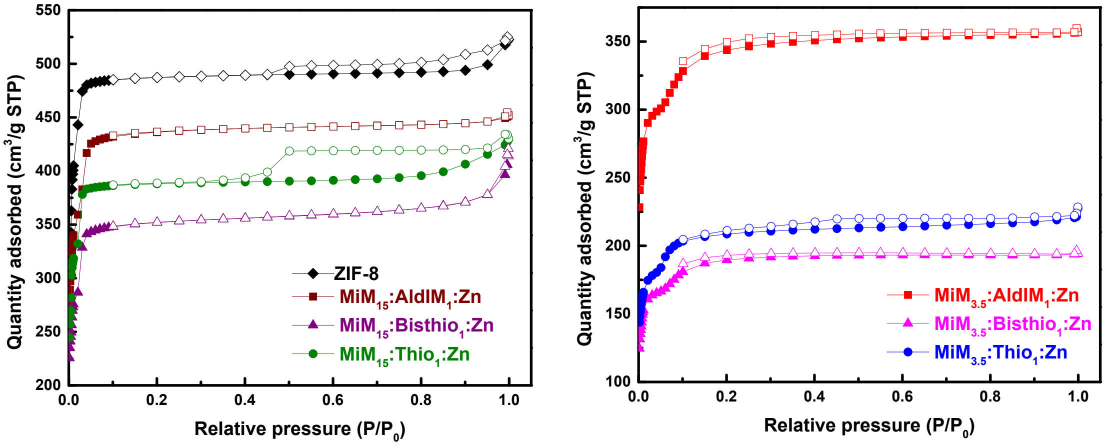

Figure S13: N_2 adsorption (filled circles) and desorption (empty circles) isotherms at 77 K of **ZIF-8**, **Ald-ZIF** and **TSC-ZIF**

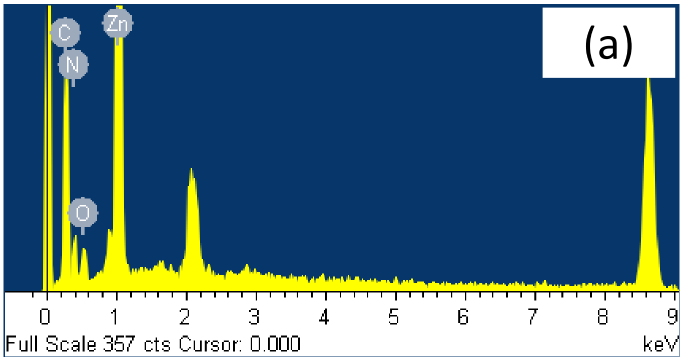

Element	Weight%	Atomic%
C K	42.40	54.32
N K	28.47	31.28
O K	10.38	9.98
Zn K	18.76	4.42
Totals	100.00	

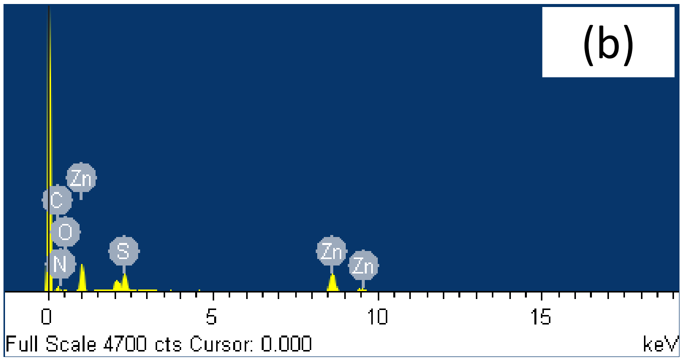

Element	Weight%	Atomic%
C K	31.92	46.73
N K	25.15	31.58
O K	9.92	10.90
S K	6.84	3.75
Zn K	26.18	7.04
Totals	100.00	

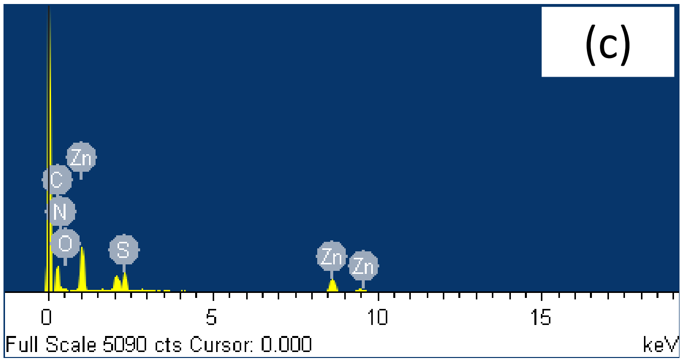

Element	Weight%	Atomic%
C K	44.04	53.33
N K	34.04	35.35
O K	8.42	7.65
S K	2.89	1.31
Zn K	10.61	2.36
Totals	100.00	

Figure S14: EDX spectra of SEM images for (a) **$MIM_{3.5}$:$AldIM_1$:Zn**, (b) **$MIM_{3.5}$:$Bisthio_1$:Zn** and (c) **$MIM_{3.5}$:$Thio_1$:Zn**

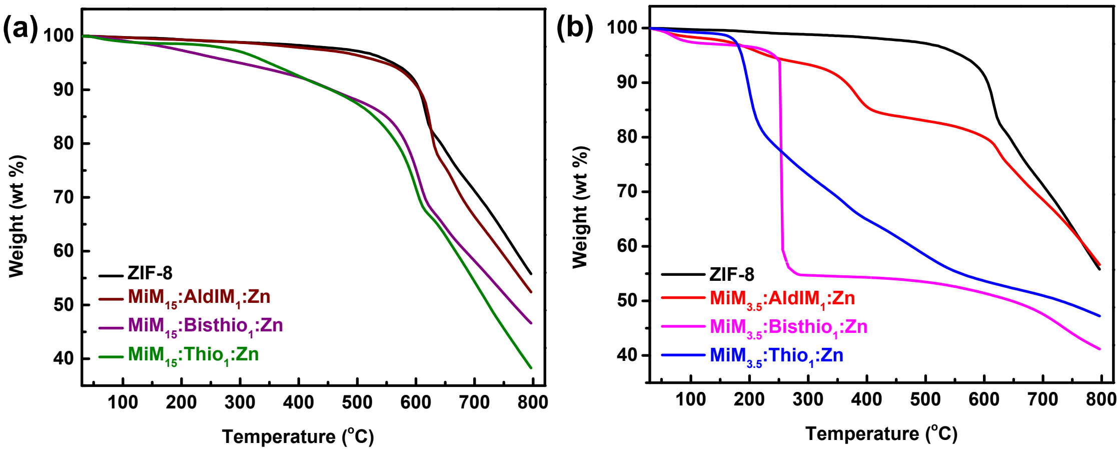

Figure S15: Thermogravimetric analysis of **ZIF-8**, **Ald-ZIF** and **TSC-ZIF**

3. Hg^{2+} removal efficiency from water

3.1. Kinetic experiments

To explore the percentage removal and the kinetic mechanism of **ZIF-8**, **MIM_{15}:AldIM$_1$:Zn, MIM_{15}:Bisthio$_1$:Zn** and **MIM_{15}:Thio$_1$:Zn**, were investigated through suspending 60 mg of each MOF in 60 mL of 100 ppm Hg^{2+} ions solution (pH = 7), then the suspension was placed on a platform shaker and shaken at 350 rpm at room temperature. At predetermined time intervals (30, 60, 120, 240, 1440 min), 10 mL aliquot of the suspension was filtered through a 200 nm membrane filter, then the remaining Hg^{2+} ions content in filtrates was analyzed by AAS

To explore the percentage removal and the kinetic mechanism of **$MIM_{3.5}$:AldIM$_1$:Zn, $MIM_{3.5}$:Bisthio$_1$:Zn** and **$MIM_{3.5}$:Thio$_1$:Zn**, were investigated through suspending 60 mg of each MOF in 60 mL of 400 ppm Hg^{2+} ions solution (pH = 7), then the suspension was placed on a platform shaker and shaken at 350 rpm at room temperature. At predetermined time intervals (30, 60, 120, 240, 1440 min), 10 mL aliquot of the suspension was filtered through a 200 nm membrane filter, then the remaining Hg^{2+} ions content in filtrates was analyzed by AAS

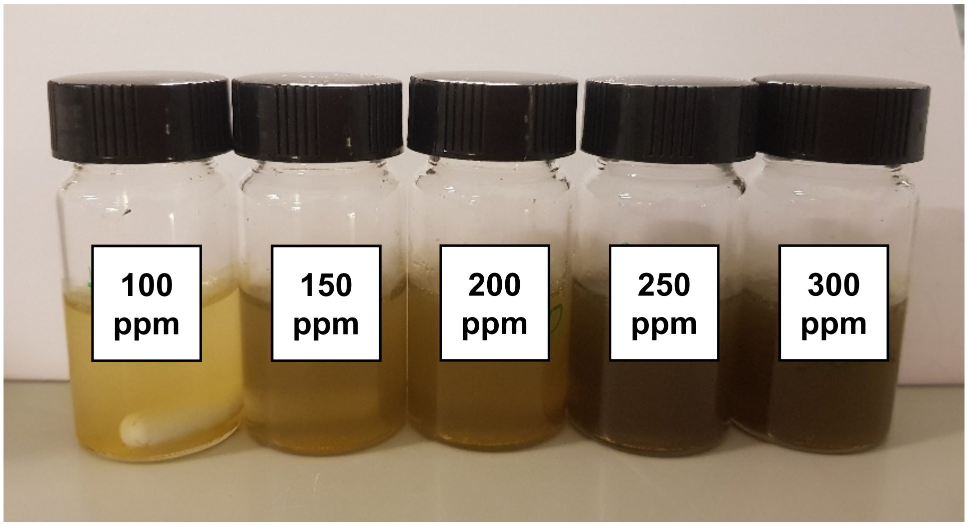

Figure S16: Pictures of **$MIM_{3.5}$:Thio:Zn (originally yellow in colour)** solutions with different concentrations of Hg^{2+} solutions (colourless) The gray-black colour of the **$MIM_{3.5}$:$Thio_1$:Zn**. indicates the adsorption of Hg^{2+}.

3.1.1. Percentage removal

The metal uptake per gram of sorbent and the percentage removal was calculated according to the following equations (eqn S1 and S2) [3]:

$$Removal\ (\%) = \frac{(Ci - Ce)\ \times\ 100}{Ci} \qquad \text{(S1)}$$

$$qe = \frac{(\mathrm{Ci} - \mathrm{Ce})\ \times\ \mathrm{V}}{\mathrm{w}} \qquad \text{(S2)}$$

Where: $\boldsymbol{q_e}$ is the equilibrium adsorption capacity (mg/g), $\boldsymbol{C_i}$ is the initial concentration (mg/L) of metal ions in solution, $\boldsymbol{C_e}$ was the equilibrium concentration (mg/L) of metal ions in solution, $\boldsymbol{V}$ is the volume of aqueous solution (L) and $\boldsymbol{m}$ is the dry weight of the adsorbent (g).

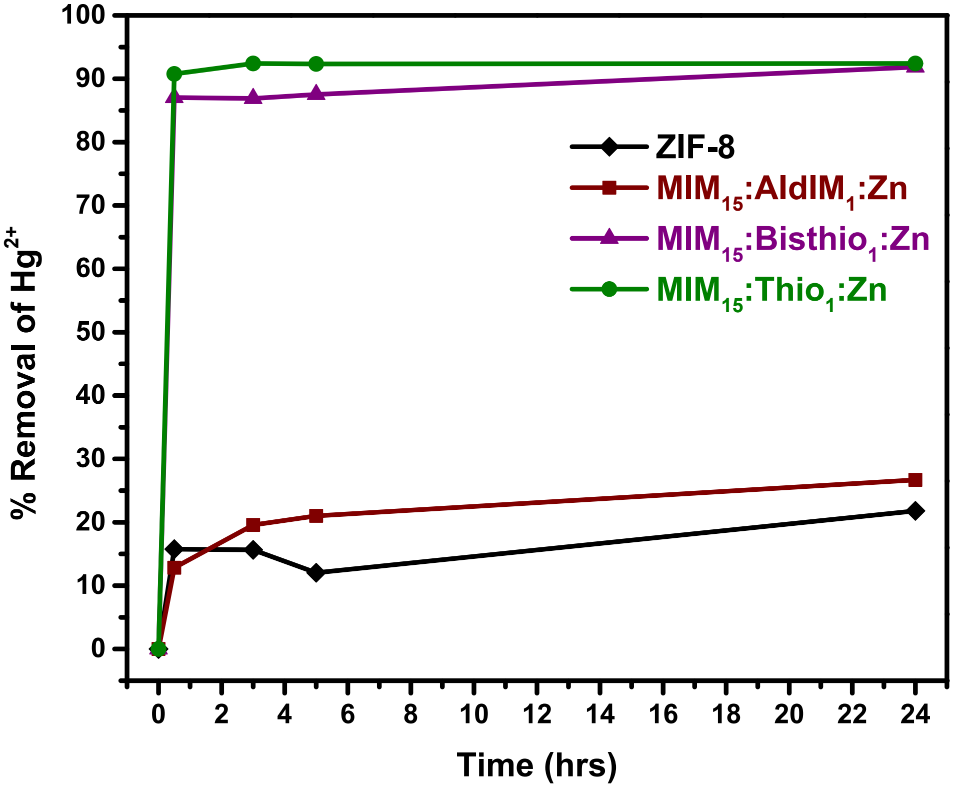

Figure S17: Percentage removal of Hg^{2+} with time using **ZIF-8**, **Ald-ZIF** and **TSC-ZIF,** ([Hg^{2+}] =100mg/L)

3.2. Hg^{2+} ions sorption isotherm

Adsorption equilibrium experiments were carried out of **ZIF-8**, **MIM_{15}:AldIM$_1$:Zn**, **MIM_{15}:Bisthio$_1$:Zn**, **MIM_{15}:Thio$_1$:Zn**, **$MIM_{3.5}$:AldIM$_1$:Zn**, **$MIM_{3.5}$:Bisthio$_1$:Zn** and **$MIM_{3.5}$:Thio$_1$:Zn** by varying the initial concentrations of the Hg^{2+} solution, when 10.0 mg of each MOF was dispersed to 10 mL Hg^{2+} ions solution (pH = 7) of different concentrations. The suspensions were shaken at 350 rpm at room temperature for 24 h and then were filtered separately through a 200 nm membrane filter. The remaining Hg^{2+} ions content in filtrates was analyzed by AAS.

3.2.1. Adsorption isotherm models

Two traditionally used isotherms including Langmuir and Freundlich models [3] were employed to quantify and compare the performance of the **ZIF-8**, **MIM_{15}:$AldIM_1$:Zn**, **MIM_{15}:$Bisthio_1$:Zn**, **MIM_{15}:$Thio_1$:Zn**, **$MIM_{3.5}$:$AldIM_1$:Zn**, **$MIM_{3.5}$:$Bisthio_1$:Zn** and **$MIM_{3.5}$:$Thio_1$:Zn**.

Langmuir model is presented as the following equation [3] (eqn S3):

$$\frac{C_e}{q_e} = \frac{1}{K_L q_m} + (\frac{1}{q_m})C_e \qquad \text{(S3)}$$

Where **C_e** (mg/L) is the equilibrium concentration of Hg^{2+} solution, **q_e** represents the adsorption capacity of Hg^{2+} at equilibrium (mg/g), **k_L** is the Langmuir constant (L/mg) related to adsorption energy and affinity of binding sites, and **q_m** denotes the maximum adsorption capacity (mg/g). The values of **q_m** and **K_L** can be determined from the linear plot of **C_e/q_e** versus **C_e**

The essential characteristics of the Langmuir isotherm parameters can be used to predict the affinity between the sorbate and the sorbent using the separation factor or dimensionless equilibrium parameter 'R_L', expressed as in the following equation (eqn S4): [3]

$$R_L = \frac{1}{1 + K_L C_0} \qquad \text{(S4)}$$

Where: K_L is the Langmuir constant and C_0 is the initial concentration of the metal ion. The value of the separation factor R_L provides important information about the nature of adsorption. The value of R_L is between 0 and 1 for favorable adsorption, while $R_L>1$ represents unfavorable adsorption and $R_L=1$ represents linear adsorption. The adsorption process is irreversible if $R_L= 0$.

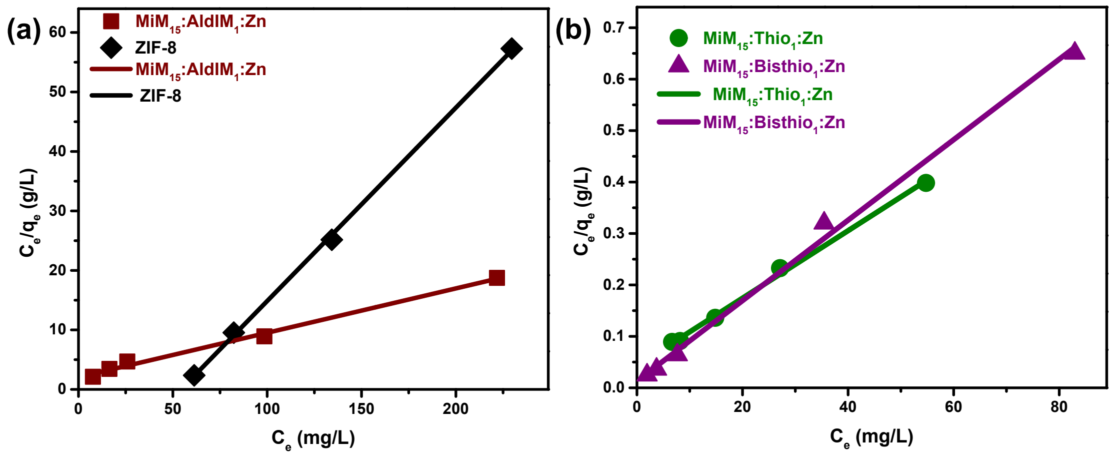

Figure S18: Langmuir isotherm plots for: (a) **ZIF-8** and **MIM_{15}:AldIM$_1$:Zn**, (b) **MIM_{15}:Bisthio$_1$:Zn**, and **MIM_{15}:Thio$_1$:Zn**

Freundlich adsorption equation (Eqn S5) has the linear form [3] as follows:

$$Log\ q_e = Log\ K_F + \left(\frac{1}{n}\right) Log\ C_e \qquad (S5)$$

Where: q_e is the metal uptake (mg/g) at equilibrium, K_F is the measure of the sorption capacity, $1/n$ is the sorption intensity, and C_e is the final ion concentration in solution, or equilibrium concentration (mg/L). The Freundlich isotherm constants K_F and $1/n$ are evaluated from the intercept and the slope, respectively of the linear plot of log q_e versus log C_e.

The value of **n** reflects the type of isotherm to be favorable ($0 < 1/n < 1$), irreversible ($1/n = 0$) or unfavorable ($1/n > 1$).[3]

Table S1: Freundlich equation parameters for Hg^{2+} ions adsorption onto the as-prepared **ZIF-8**, **Ald-ZIF** and **TSC-ZIF**

Freundlich models			
**Samples**	K_F (L/mg)	1/**n**	R^2
**ZIF-8**	0.0045	0.74	0.9707
**MiM_{15}:AldIM$_1$:Zn**	1.66	0.38	0.9712
**MiM_{15}:Bisthio$_1$:Zn**	73.69	0.10	0.8084
**MiM_{15}:Thio$_1$:Zn**	49.4	0.26	0.9237
**$MiM_{3.5}$:AldIM$_1$:Zn**	33	0.23	0.8331
**$MiM_{3.5}$:Bisthio$_1$:Zn**	365	0.2	0.9692
**$MiM_{3.5}$:Thio$_1$:Zn**	157	0.38	0.9593

3.2.2. Adsorption Kinetic

The kinetic data were fitted with the pseudo-second-order kinetic model using the following equation [4] (eqn S6):

$$\frac{t}{qt} = \frac{1}{K_2 q_e^2} + \frac{t}{q_e} \qquad \text{(S6)}$$

Where $\mathbf{q_e}$ (mg/g) and $\mathbf{q_t}$ (mg/g) are the amount of Hg^{2+} ions adsorbed at equilibrium and at time **t** (min), respectively; **t** (min) is adsorption time and $\mathbf{k_2}$ (g/mg/min) is the pseudo-second-order adsorption rate constant.

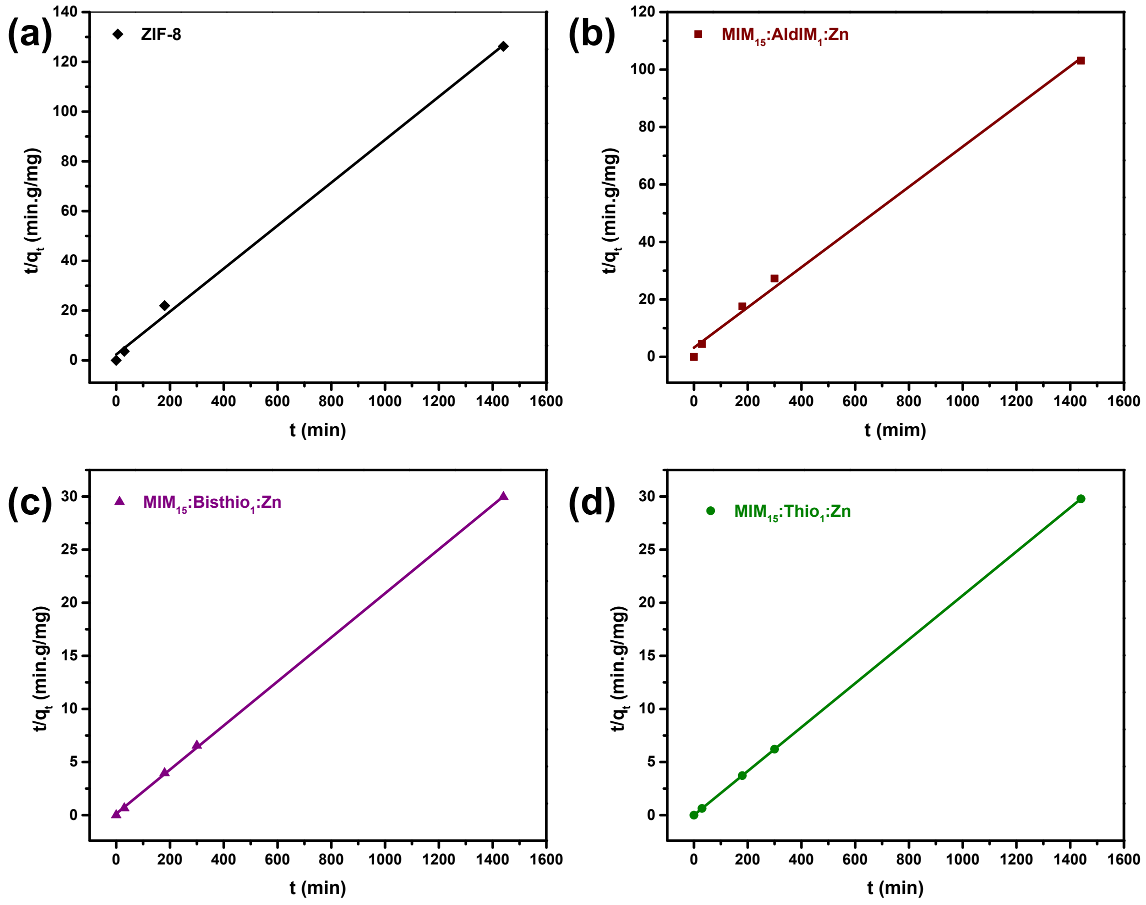

Figure S19: The pseudo-second order kinetic plot for the adsorption ($[Hg^{2+}]_i$ = 100 ppm) for: (a) **ZIF-8**, (b) **MIM$_{15}$*:*Ald$_1$:Zn**, (c) **MIM$_{15}$*:*Bithio$_1$:Zn** and (d) **MIM$_{15}$:Thio$_1$:Zn**

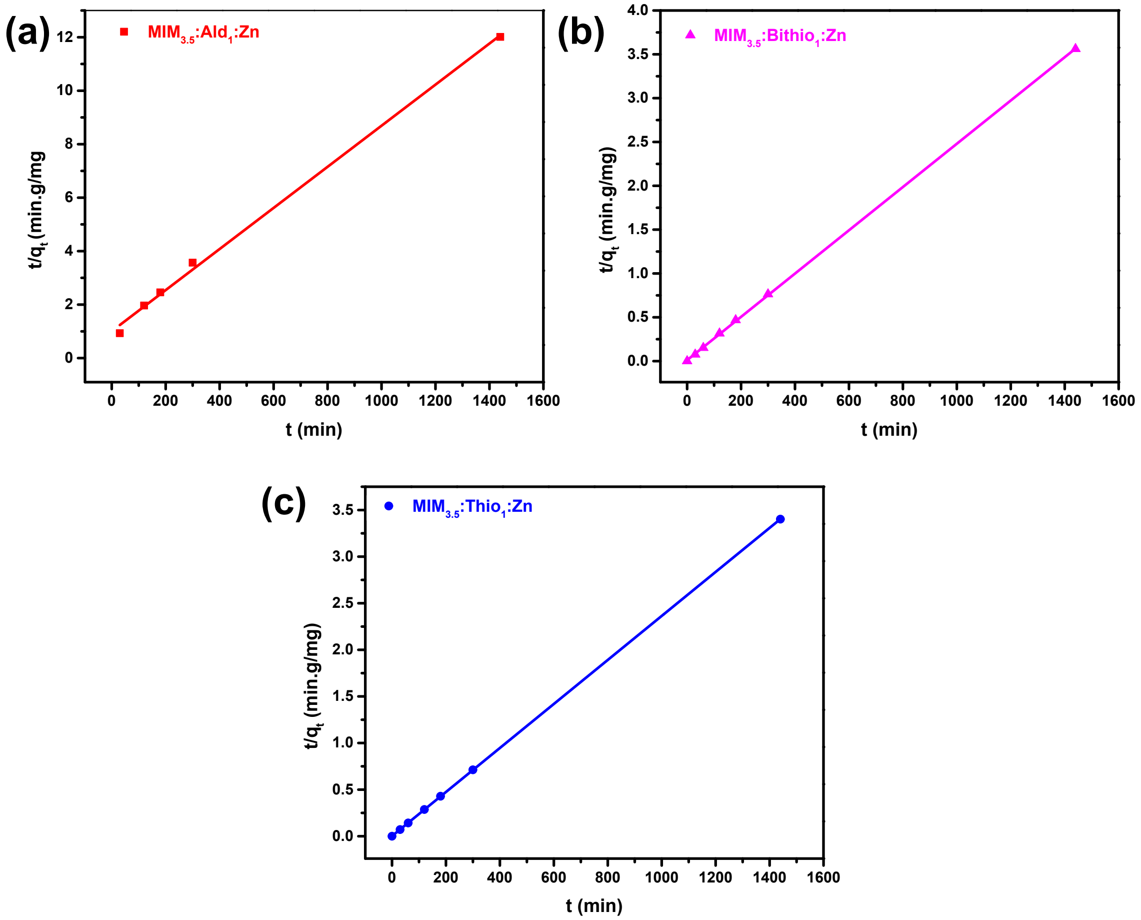

Figure S20: The pseudo-second order kinetic plots for the adsorption ([Hg^{2+}]=400 ppm) for : (a) **$MIM_{3.5}$:Ald_1:Zn**, (b) **$MIM_{3.5}$:$Bithio_1$:Zn** and (c) **$MIM_{3.5}$:$Thio_1$:Zn**

Table S20: Pseudo-second order equation parameters for Hg^{2+} ions adsorption onto the as-prepared **ZIF-8**, **Ald-ZIF** and **TSC-ZIF**

**Pseudo-second order model**		
**Samples**	K_2 (g/mg/min)	R^2
**ZIF-8**	9 x10^{-3}	0.9977
**MIM_{15}:$AldIM_1$:Zn**	4 x 10^{-3}	0.9965

**MIM_{15}:$Bisthio_1$:Zn**	1×10^{-2}	0.9999
**MIM_{15}:$Thio_1$:Zn**	2.9×10^{-1}	1
**$MIM_{3.5}$:$AldIM_1$:Zn**	0.59×10^{-4}	0.9979
**$MIM_{3.5}$:$Bisthio_1$:Zn**	0.55×10^{-3}	0.9999
**$MIM_{3.5}$:$Thio_1$:Zn**	0.32×10^{-2}	1

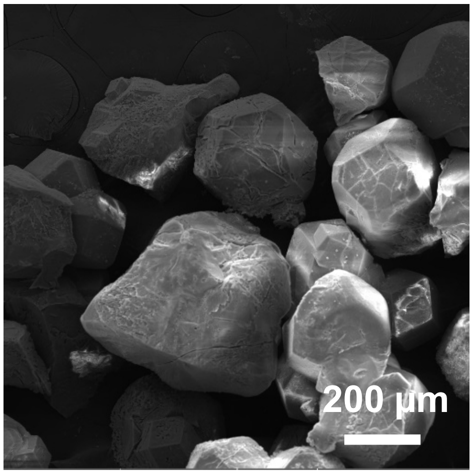

Figure S21: SEM images for **$MIM_{3.5}$:$Thio_1$:Zn** after adsorption of Hg^{2+}

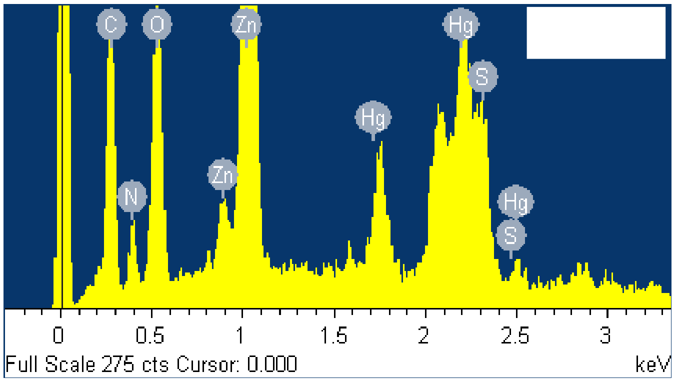

Element	Weight%	Atomic%
C K	23.85	44.03
N K	10.18	16.12
O K	19.35	26.82
S K	1.44	1.00
Zn L	30.74	10.43
Hg M	14.45	1.60

Figure S22: EDX spectra of SEM images for the **$MIM_{3.5}$:$Thio_1$:Zn** after the adsorption of Hg^{2+}

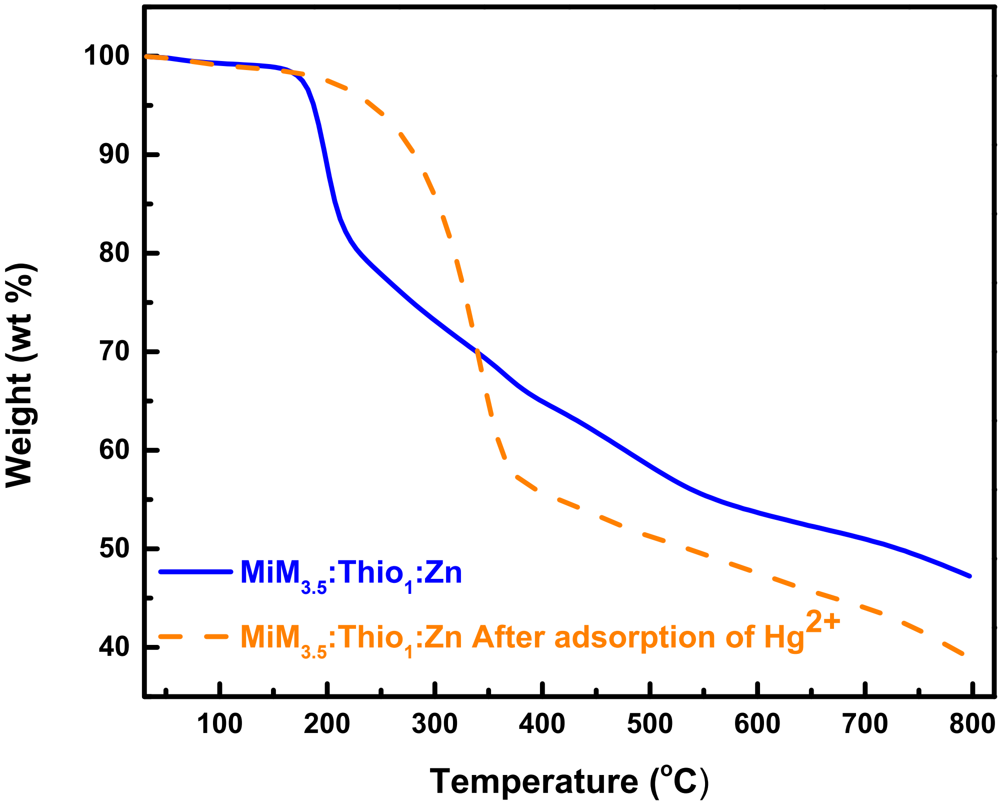

Figure S23: TGA profile of the **$MIM_{3.5}$:$Thio_1$:Zn** before and after adsorption of Hg^{2+}

3.3. Ion selectivity tests

3.3.1. Competitive binding: binary system (Hg^{2+} and Pb^{2+})

The effect of Pb^{2+} ions, on the removal efficiency of Hg^{2+} ions **$MIM_{3.5}$:$Thio_1$:Zn** was studied. The initial concentration of Hg^{2+} in the metal solutions was varied from 100 to 400 ppm by keeping Pb^{2+}, concentration constant ([Pb^{2+}] = 1000 ppm)

10.0 mg of **$MIM_{3.5}$:$Thio_1$:Zn** was dispersed in 10 mL of metals solutions (pH =7). The suspensions were shaken at 350 rpm at room temperature for 12 h and then filtered separately through a 200 nm membrane filter. The remaining ions content in filtrates were analyzed by AAS.

3.3.2. Competitive binding: tertiary system (Hg^{2+}, Pb^{2+} and Cd^{2+})

The effect of different co-ions such as Pb^{2+} and Cd^{2+}, which are commonly present in wastewater, on the removal efficiency of Hg^{2+} ions **$MIM_{3.5}$:$Thio_1$:Zn** was studied. The initial concentration of Hg^{2+} in the metal solutions was varied from 100 to 400 ppm by keeping Pb^{2+} and Cd^{2+} concentration constant (each metal 1000 ppm). 10.0 mg of **$MIM_{3.5}$:$Thio_1$:Zn** was dispersed in 10 mL of metals solutions (pH =7). The suspensions were shaken at 350 rpm at room temperature for 12 h and then filtered separately through a 200 nm membrane filter. The remaining ions content in filtrates were analyzed by AAS.

4. Regeneration of $MIM_{3.5}$:$Thio_1$:Zn

The potential reusability of **$MIM_{3.5}$: $Thio_1$: Zn** was also tested by the TSC-ZIF with *p*-toluene sulfonic acid (0.1 M). **$MIM_{3.5}$:$Thio_1$:Zn**, 8 mg, was dispersed in 10 mL aqueous solution containing 700 ppm Hg^{2+} (pH = 7), the suspension was then placed on a shaker set at 350 rpm at room temperature, for 24 h. The mixture was then separated by centrifugation. Mercury removal was assessed by analyzing its content in the supernatant using AAS, whereas the solid (Hg^{2+} adsorbed on the TSC-ZIF) was treated with 10 mL *p*-toluene sulfonic acid (0.1 M) solution (pH=4) for 2 h to regenerate the adsorbent. The regenerated TSC-ZIF was washed three times with water to be used in another cycle of Hg^{2+} removal. This process was repeated five time.

References and notes

[1] Gaussian 09, Revision E.01, M. J. Frisch, G. W. Trucks, H. B. Schlegel, G. E. Scuseria, M. A. Robb, J. R. Cheeseman, G. Scalmani, V. Barone, G. A. Petersson, H. Nakatsuji, X. Li, M. Caricato, A. Marenich, J. Bloino, B. G. Janesko, R. Gomperts, B. Mennucci, H. P. Hratchian, J. V. Ortiz, A. F. Izmaylov, J. L. Sonnenberg, D. Williams-Young, F. Ding, F. Lipparini, F. Egidi, J. Goings, B. Peng, A. Petrone, T. Henderson, D. Ranasinghe, V. G. Zakrzewski, J. Gao, N. Rega, G. Zheng, W. Liang, M. Hada, M. Ehara, K. Toyota, R. Fukuda, J. Hasegawa, M. Ishida, T. Nakajima, Y. Honda, O. Kitao, H. Nakai, T. Vreven, K. Throssell, J. A. Montgomery, Jr., J. E. Peralta, F. Ogliaro, M. Bearpark, J. J. Heyd, E. Brothers, K. N. Kudin, V. N. Staroverov, T. Keith, R. Kobayashi, J. Normand, K. Raghavachari, A. Rendell, J. C. Burant, S. S. Iyengar, J. Tomasi, M. Cossi, J. M. Millam, M. Klene, C. Adamo, R. Cammi, J. W. Ochterski, R. L. Martin, K. Morokuma, O. Farkas, J. B. Foresman, and D. J. Fox, Gaussian, Inc., Wallingford CT, 2016

[2] A. D. Becke, *J. Chem. Phys.*, 1993, **98**, 5648–5652. C. Lee, W. Yang and R. G. Parr, *Phys. Rev. B*, 1988, **37**, 785–789.

[3] Mahmoud, M. E.; Amira, M. F.; Seleim, S. M.; Mohamed, A. K. Adsorption Isotherm Models, Kinetics Study, and Thermodynamic Parameters of Ni(II) and Zn(II) Removal from Water Using the LbL Technique. *J. Chem. Eng. Data* **2017**, *62* (2), 839–850. https://doi.org/10.1021/acs.jced.6b00865.

[4] Yang, P.; Shu, Y.; Zhuang, Q.; Li, Y.; Gu, J. A Robust MOF-Based Trap with High-Density Active Alkyl Thiol for the Super-Efficient Capture of Mercury. *Chem. Commun.* **2019**, *55* (86), 12972–12975. https://doi.org/10.1039/C9CC06255F.
